# Central role for p62/SQSTM1 in the elimination of toxic tau species in a mouse model of tauopathy

**DOI:** 10.1111/acel.13615

**Published:** 2022-06-05

**Authors:** Maiko Ono, Masaaki Komatsu, Bin Ji, Yuhei Takado, Masafumi Shimojo, Takeharu Minamihisamatsu, Eiji Warabi, Toru Yanagawa, Gen Matsumoto, Ichio Aoki, Nicholas M. Kanaan, Tetsuya Suhara, Naruhiko Sahara, Makoto Higuchi

**Affiliations:** ^1^ Quantum Life and Medical Science Directorate National Institutes for Quantum Science and Technology Chiba Japan; ^2^ Department of Physiology Juntendo University Graduate School of Medicine Tokyo Japan; ^3^ Department of Radiopharmacy and Molecular Imaging Fudan University Shanghai China; ^4^ 13121 Faculty of Medicine University of Tsukuba Tsukuba Japan; ^5^ Department of Histology and Cell Biology Nagasaki University Graduate School of Biomedical Sciences Nagasaki Japan; ^6^ 3078 Department of Translational Neuroscience College of Human Medicine Michigan State University Grand Rapids Michigan USA

**Keywords:** autophagy, FTDP‐17, p62/SQSTM1, tau, tau oligomers, tauopathy model mouse

## Abstract

Intracellular accumulation of filamentous tau aggregates with progressive neuronal loss is a common characteristic of tauopathies. Although the neurodegenerative mechanism of tau‐associated pathology remains unclear, molecular elements capable of degrading and/or sequestering neurotoxic tau species may suppress neurodegenerative progression. Here, we provide evidence that p62/SQSTM1, a ubiquitinated cargo receptor for selective autophagy, acts protectively against neuronal death and neuroinflammation provoked by abnormal tau accumulation. P301S mutant tau transgenic mice (line PS19) exhibited accumulation of neurofibrillary tangles with localization of p62 mostly in the brainstem, but neuronal loss with few neurofibrillary tangles in the hippocampus. In the hippocampus of PS19 mice, the p62 level was lower compared to the brainstem, and punctate accumulation of phosphorylated tau unaccompanied by co‐localization of p62 was observed. In PS19 mice deficient in p62 (PS19/p62‐KO), increased accumulation of phosphorylated tau, acceleration of neuronal loss, and exacerbation of neuroinflammation were observed in the hippocampus as compared with PS19 mice. In addition, increase of abnormal tau and neuroinflammation were observed in the brainstem of PS19/p62‐KO. Immunostaining and dot‐blot analysis with an antibody selectively recognizing tau dimers and higher‐order oligomers revealed that oligomeric tau species in PS19/p62‐KO mice were significantly accumulated as compared to PS19 mice, suggesting the requirement of p62 to eliminate disease‐related oligomeric tau species. Our findings indicated that p62 exerts neuroprotection against tau pathologies by eliminating neurotoxic tau species, suggesting that the manipulative p62 and selective autophagy may provide an intrinsic therapy for the treatment of tauopathy.

## Graphical abstract

This study proposes a mechanism that p62/SQSTM1, a ubiquitinated cargo receptor for selective autophagy, acts protectively against neuronal death and neuroinflammation as tau pathologies by eliminating disease‐related oligomeric tau formed during the aggregation process of abnormal tau.

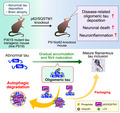

## 1 INTRODUCTION

Abnormal tau accumulation is implicated in neurodegenerative diseases, including Alzheimer's disease (AD), progressive supranuclear palsy (PSP), corticobasal degeneration (CBD), Pick's disease (PiD), and frontotemporal dementia with Parkinsonism linked to chromosome 17 (FTDP‐17), which are collectively known as tauopathies (Lee et al., 2001). Intracellular filamentous tau aggregates are closely associated with the severity of brain dysfunction and neuronal loss (Arriagada et al., 1992), and therefore, approaches to attenuating tau aggregation can be a major therapeutic strategy for the treatment of several neurodegenerative diseases (Jadhav et al., 2019). Recent studies demonstrated that the degradation system of the autophagy‐lysosome pathway via specific cargo receptors, such as p62/SQSTM1 (p62), selectively suppresses the accumulation of tau aggregates (Babu et al., 2008; Xu et al., 2019). The multifunctional protein p62 contains a ubiquitin‐binding domain, an LC3‐interacting region, and a Phox and Bem1 (PB1) domain mediating self‐polymerization (Katsuragi et al., 2015). p62 is one of the key mediators of selective autophagy in cytosol, which recruits ubiquitinated protein aggregates to the autophagic degradation process (Komatsu et al., 2007). In addition, p62 also packages ubiquitinated proteins into inclusion bodies as it self‐assembles (Moscat et al., 2007). Abnormally phosphorylated tau in AD brains is ubiquitinated (Mori et al., 1987), and immunohistochemical examinations have confirmed a localization of p62 with tau inclusions in human tauopathies (Scott & Lowe, 2007). Thus, p62 and autophagy are presumed to be involved in the degradation and sequestration of this pathological form of tau, and they may be crucial for the homeostatic protection of neurons against tau‐provoked toxicities.

Animal models of tauopathies are essential for preclinical studies of AD and related neurodegenerative diseases. Overexpression of wild‐type tau transgenes has produced only limited success in modeling the mature pathology observed in the human tauopathies (Jankowsky & Zheng, 2017). Most mouse modeling approaches express FTDP‐17‐associated tau mutations to accelerate tau self‐assembly. Among them, the P301S mutant human tau transgenic mouse expressing 1N4R tau under control of the mouse prion promoter (PS19 mouse line) (Yoshiyama et al., 2007) is widely utilized. In this mouse model, filamentous tau inclusions resembling neurofibrillary tangles (NFTs) are abundantly developed in the brainstem and spinal cord. A unique characteristic of this model is extensive neuronal death prior to the formation of NFT‐like inclusions in the hippocampus.

We hypothesized that p62 has pivotal roles in the degradation and sequestration of toxic tau species in a mouse model of tauopathy. To test this hypothesis, we generated double‐mutant PS19/p62‐KO mice and investigated the accumulation of abnormal tau species, the extent of neuronal loss, and the degree of neuroinflammation in those mice compared to PS19 mice.

## 2 RESULTS

### 2.1 Involvement of p62 with various tau pathologies in human tauopathies and PS19 mice

**FIGURE 1:**
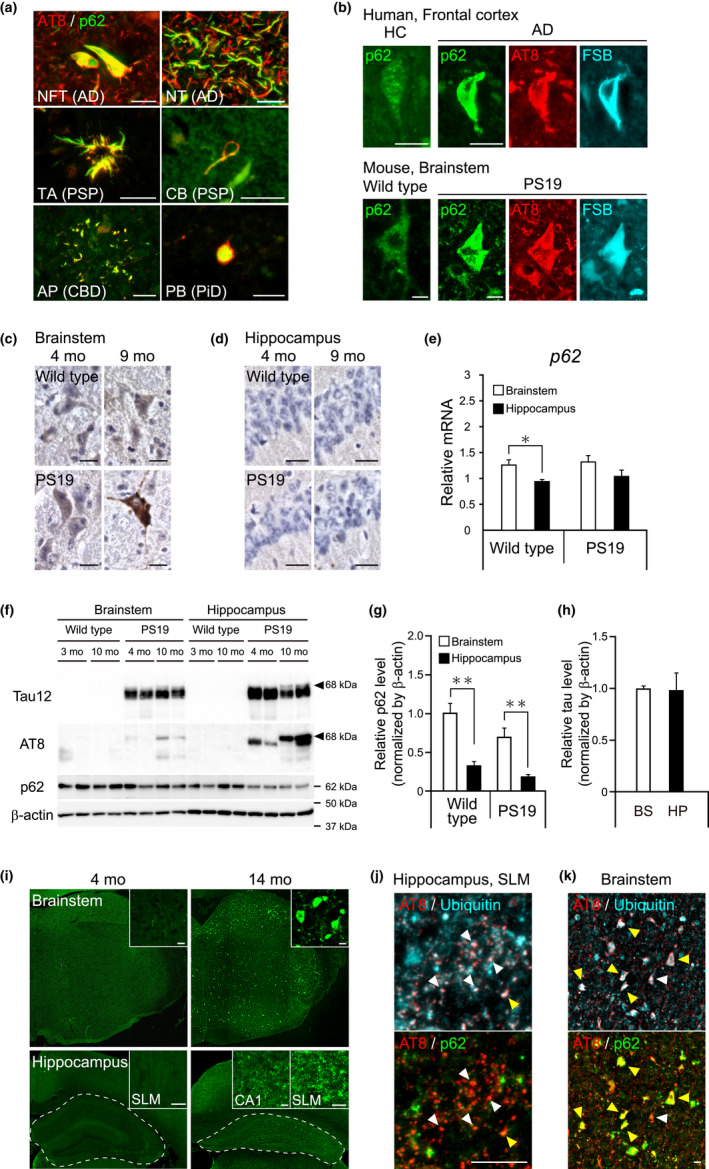
Involvement of p62 with various tau pathologies in human tauopathies and PS19 mice. (a) Double‐labeling of anti‐phospho‐tau antibody (AT8, red) and anti‐p62 antibody (green) confirmed co‐localization of p62 with neurofibrillary tangles (NFT) and neuropil threads (NT) in AD brains, tufted astrocytes (TA) and coiled bodies (CB) in PSP brains, astrocytic plaques (AP) in CBD brains, and pick bodies (PB) in PiD brain. (b) p62 (green) coexisted with FSB‐positive (blue) phosphorylated (red) tau inclusions in neurons of AD (upper panels) and PS19 mouse (lower panels) brains. In contrast to tangle‐bearing neurons, punctate staining of p62 antibody was observed in neurons of human healthy control (HC) (upper panel) and wild‐type mouse (lower panel). (c, d) Immunoreactivity of p62 in brainstem (c) and hippocampus (d) of 4‐ and 9‐month‐old (mo) wild‐type and PS19 mice. Staining was visualized by 3,3′‐diaminobenzidine (DAB) chromogen (brown color). Immunoreactivity of p62 was weak or almost absent in CA1 region of these mice. (e) Quantitative real‐time PCR analysis of p62 mRNA in brainstem and hippocampus of 8‐month‐old wild‐type (*n* = 5) and PS19 mice (*n* = 5). (f) Western blotting of wild‐type and PS19 mouse brains labeled by anti‐tau (Tau12), anti‐phospho‐tau (AT8), anti‐p62, and anti‐β‐actin antibodies. TBS‐soluble fractions from brainstems and hippocampi of 3‐month‐old (*n* = 2) and 10‐month‐old (*n* = 2) wild‐type and 4‐month‐old (*n* = 2) and 10‐month‐old (*n* = 2) PS19 mice were separated in SDS‐PAGE. Tau12 and AT8 blots showed hyperphosphorylated 68 kDa tau bands (arrowheads) in 10‐month‐old PS19 brains. (g) p62‐positive signal in Western blotting was compared between brainstem and hippocampus in wild‐type and PS19 mice. The relative p62 level was normalized by β‐actin signal. The averaged signal in brainstem of wild‐type mice was set as 1. (h) Tau12‐positive signal in Western blotting was compared between brainstem (BS) and hippocampus (HP) of PS19 mice. The relative tau level was normalized by β‐actin signal. The averaged signal in brainstem was set as 1. (i) Coronal sections of brainstem (upper panels) and hippocampus (lower panels) from 4‐ and 14‐month‐old (mo) PS19 mice were labeled by AT8 antibody. Inserts show high‐magnification views. White dash lines enclose the hippocampus region. In aged PS19 mice, AT8‐positive intracellular inclusions were abundantly detected in the brainstem (upper inserts), whereas these tau inclusions were absent in hippocampal cell soma (lower CA1 inserts). On the contrary, intense accumulation of granular dot‐like aggregates of phosphorylated tau was observed in stratum lacunosum‐moleculare of hippocampus (lower SLM insert). (j, k) Stratum lacunosum‐moleculare of hippocampus (j) and brainstem (k) from 14‐month‐old PS19 mice were triple‐immunolabeled with AT8 (red), anti‐ubiquitin (blue), and anti‐p62 (green) antibodies. White arrowheads represent merging of phosphorylated tau and ubiquitin, but not p62, immunoreactivity. Yellow arrowheads represent triple merging of phosphorylated tau, ubiquitin, and p62 immunoreactivity. Scale bars, 20 μm. Data are shown as mean ± SD. *p < 0.05, **p < 0.005 by Welch's *t*‐test

To examine whether p62 is a common component of a broad range of tau pathologies, brain sections from various human tauopathies were immunolabeled with antibodies against tau and p62 proteins. p62 predominantly coexisted with phosphorylated tau in the NFTs and neuropil threads in the frontal cortex of AD, tufted astrocytes and coiled bodies in the striatum of PSP, astrocytic plaques in the frontal cortex of CBD, and Pick bodies in the frontal cortex of PiD patients (Figure 1a, Table S1). The punctate immunoreactivity of p62 was found in neuronal somas in the hippocampi of healthy control subjects and the brainstems of wild‐type mice (Figure 1b). The punctate immunoreactivity of p62 was also found in neuronal somas without accumulation of phosphorylated tau in the hippocampi of AD patients and the brainstems of PS19 mice (Figure S1). Remarkably, the immunoreactivity of p62 was robustly intensified with accumulation of filamentous tau aggregates recognized by β‐sheet ligand FSB in AD patients and the brainstems of PS19 mice (Figure 1b). In contrast to neurons in the brainstem, immunoreactivity of p62 was barely detected in hippocampal CA1 neurons of wild‐type and PS19 mice (Figure 1c,d). The mRNA level of *p62* gene was significantly lower in the hippocampus relative to the brainstem of wild‐type mice (*p* = 0.021), and a similar trend was observed in PS19 mice (Figure 1e). To examine the spatiotemporal protein distribution of both p62 and tau in PS19 mice, Western blotting of Tris‐buffer saline (TBS)‐soluble and sarkosyl‐insoluble fractions was performed. The p62 protein was detected in the TBS‐soluble fraction (Figure S2a). In accordance with the mRNA levels, quantitative analysis of the p62 protein showed a significantly lower level in the hippocampus than in the brainstem (Figure 1f,g: wild type: *p* = 0.0018; PS19: *p* = 0.0035). Prion promoter‐regulated human P301S tau was distributed in the whole brain of PS19 mice, with similar expression levels in the hippocampus and brainstem (Figure 1f,h: *p* = 0.0162, Figure S2a), whereas amounts of hyperphosphorylated tau in TBS‐soluble fraction from the hippocampus were profoundly increased with age and higher than in that fraction from the brainstem (Figure 1f). Sarkosyl‐insoluble hyperphosphorylated 68 kDa tau was recovered in the brainstem and hippocampus of 10‐month‐old PS19 mice but not in the brains of 4‐month‐old PS19 mice (Figure S2b). PS19 mice developed age‐dependent intra‐neuronal tau deposits like NFTs in the brainstem (Figure 1i, top). In contrast, NFT‐like tau inclusions were hardly detected in the hippocampus, whereas diffuse tau aggregates were increased with aging instead (Figure 1i, bottom). In the stratum lacunosum‐moleculare of the hippocampus of older (14 months) PS19 mice, granular dot‐like aggregates of phosphorylated tau had accumulated abundantly. These phosphorylated tau immunoreactivities were co‐localized with ubiquitin (Figure 1j, white arrowheads), but the triple co‐localization of phosphorylated tau, ubiquitin, and p62 was rarely observed (Figure 1j, yellow arrowheads, Figure S3). In contrast, co‐localizations of phosphorylated tau, ubiquitin, and p62 were frequently observed in NFT‐like inclusions in the brainstem (Figure 1k and Figure S3). Consistent with previous studies (Yoshiyama et al., 2007), the hippocampal size of 14‐month‐old PS19 mice was significantly reduced in comparison with that of 4‐month‐old PS19 mice (Figure 1i). Thus, massive neuronal death progressively occurred in the hippocampus prior to the formation of NFT‐like tau inclusions compared to the brainstem.

These results suggest a shortage of deleterious component processing by p62 at a particularly advanced stage of age‐dependent accumulation of phosphorylated tau due to lower basal expression of p62 and higher expression of human P301S tau in the hippocampus of PS19 mice in comparison with the brainstem.

### 2.2 Effects of p62 deficiency on abnormal tau accumulation in PS19 mice

**FIGURE 2:**
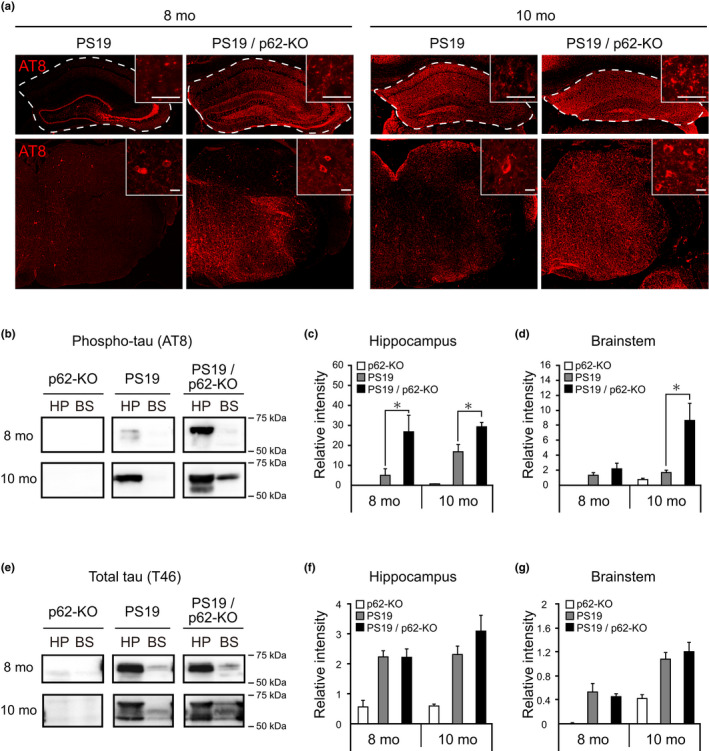
Enhancement of tau pathology in PS19 mice by genetic inactivation of p62. (a) Immunofluorescence staining of phosphorylated tau labeled by AT8 antibody in the hippocampus (upper panels) and brainstem (lower panels) of 8‐ and 10‐month‐old PS19 and PS19/p62‐KO mice. Inserts show high‐magnification views of the stratum lacunosum‐moleculare of the hippocampus (upper panels) and brainstem (lower panels). White dash lines enclose the hippocampal regions. (b–g) Total extracts from the hippocampus (HP) and brainstem (BS) of 8‐ and 10‐month‐old (mo) p62‐KO, PS19, and PS19/p62‐KO mice were separated by SDS‐PAGE and blotted with AT8 (b) and T46 (e) antibodies. Immunoblots of AT8 and T46 antibodies (*n* = 5, respectively) were measured by image quantification software. The accumulation levels of phosphorylated tau in the hippocampus of PS19/p62‐KO mice at both 8 and 10 months old (c) and the brainstem of 10‐month‐old PS19/p62‐KO mice (d) were significantly higher than those of PS19 mice, while there was no significant difference in total tau level of the hippocampus (f) or brainstem (g) between PS19 and PS19/p62‐KO. Scale bars, 20 μm. Data are presented as mean ± SEM. *p < 0.05 by one‐way ANOVA (group) followed by Tukey's HSD test at each age

The apparent involvement of p62 in the processing of phosphorylated tau directed us to examine the effects of p62 deficiency on tau pathology formation in PS19 mice. Based on the pathological characteristics of PS19 mice, the effect of p62 deficiency on tau pathology was examined. Immunostaining and biochemical analyses demonstrated a significant increase of phosphorylated tau in the hippocampus and brainstem of 8‐ to 10‐month‐old PS19/p62‐KO mice as compared to PS19 mice, while a significant accumulation of phosphorylated tau was hardly detected in the hippocampus and brainstem of p62‐KO mice after 8 months of age (Figure 2a–d and Figures S4 and S5). At the early pathological stage, 4 months of age, there was no difference in the accumulation of phosphorylated tau between PS19 and PS19/p62‐KO mice. The level of tau protein as examined by T46 antibody was not affected by the absence of p62 in the hippocampus and brainstem of PS19 mice (Figure 2e–g and Figure S5). These results suggest that p62 suppresses the accumulation of abnormal tau species without altering the expression level of tau protein in the hippocampus and brainstem of PS19 mice.

### 2.3 Effects of p62 deficiency on neuronal loss and neuroinflammation

**FIGURE 3:**
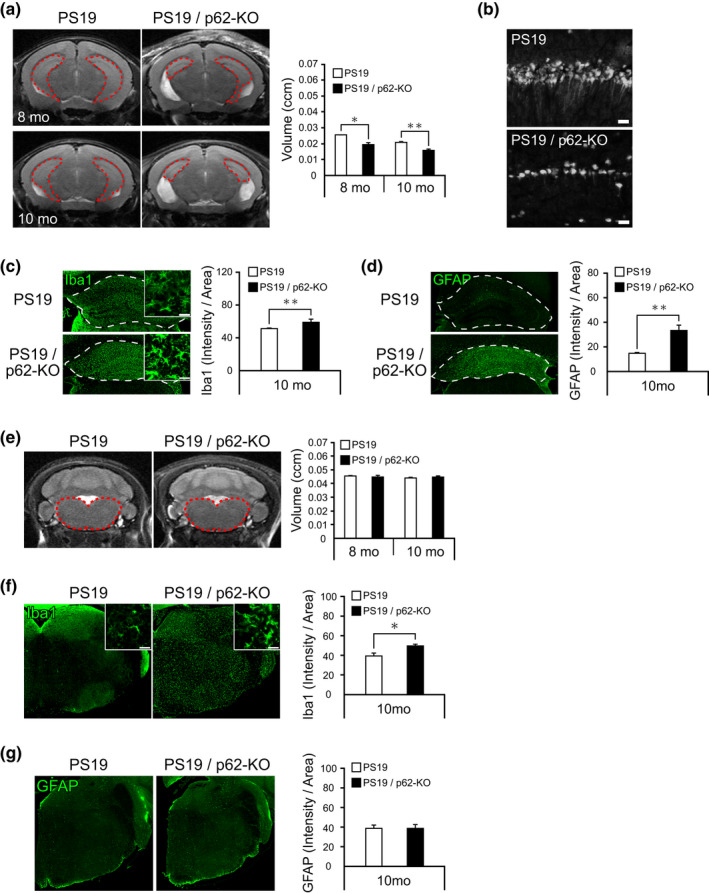
Enhancement of brain atrophy and neuroinflammation in PS19 mice by genetic inactivation of p62. (a) Regional volumes of hippocampus in 8‐ and 10‐month‐old (mo) PS19 and PS19/p62‐KO mice were quantified based on *in vivo* T2‐weighted RARE coronal MRI images (*n* = 5, respectively). Red dash lines in MR images enclose the hippocampal region. (b) The hippocampal CA1 regions from 10‐month‐old PS19 and PS19/p62‐KO mice were immunostained with anti‐NeuN antibody. (c) The hippocampi from 10‐month‐old PS19 and PS19/p62‐KO mice were immunostained with anti‐Iba1 antibody. White dash lines enclose the hippocampal region. Inserts show high‐magnification views. Signal intensities of fluorescence staining were quantified (*n* = 5, respectively). (d) The hippocampi from 10‐month‐old PS19 and PS19/p62‐KO mice were immunostained with anti‐GFAP antibody. White dash lines enclose the hippocampal region. Signal intensities of fluorescence staining were quantified (*n* = 5, respectively). (e) Regional volumes of brainstems in 10‐month‐old PS19 and PS19/p62‐KO mice were quantified based on *in vivo* T2‐weighted RARE coronal MRI images (*n* = 5, respectively). Red dash lines enclose the brainstem region. (f) The brainstems from 10‐month‐old PS19 and PS19/p62‐KO mice were immunostained with anti‐Iba1 antibody. Inserts show high‐magnification views. Signal intensities of fluorescence staining were quantified (*n* = 5, respectively). (g) The brainstems from 10‐month‐old PS19 and PS19/p62‐KO mice were immunostained with anti‐GFAP antibody. Signal intensities of fluorescence staining were quantified (*n* = 5, respectively). Scale bars, 20 μm. Data are presented as mean ± SEM. Group comparisons were performed by Welch's *t*‐test (*p < 0.05, **p < 0.005)

Since neuronal loss accompanied by neuroinflammation has been reported in the pathological brains of human tauopathies and tau transgenic mice (Leyns & Holtzman, 2017), we investigated whether p62 deficiency may affect the progress of neuronal loss and neuroinflammation in PS19 mice. *In vivo* assessment of the brain volume in living animals by magnetic resonance imaging (MRI) revealed enhanced atrophy of the hippocampus in PS19/p62‐KO mice relative to PS19 mice (Figure 3a, 8‐mo: *p* = 0.035; 10‐mo: *p* = 0.004). Postmortem assessment of brain slices demonstrated that notable neuronal loss occurred in the pyramidal cell layer of the hippocampus of a PS19/p62‐KO mouse compared to a PS19 mouse (Figure 3b). Moreover, immunofluorescence signals of the microglial marker Iba1 and the astrocytic marker GFAP were increased in the hippocampus of PS19/p62‐KO mice compared to PS19 mice (Figure 3c, Iba1: *p* = 0.002; Figure 3d, GFAP: *p* = 0.001). Immunofluorescence signals of P2Y12 receptor, which is downregulated in disease‐associated microglia (Maeda et al., 2021), were significantly decreased, those of reactive astrocyte marker C3 were increased, and in vitro binding of the TSPO PET ligand [^11^C]Ac5216 was markedly increased in the hippocampus of PS19/p62‐KO mice compared to PS19 mice (Figure S6). These data provide the first direct evidence that pathological tau accumulation due to p62 deficiency induces exacerbation of neuronal death accompanied by gliosis. Atrophy in the brainstem was not prominent in PS19/p62‐KO mice, and MRI volumes were almost identical in PS19/p62‐KO and PS19 mice at 8 and 10 months of age (Figure 3e, 8‐mo: *p* = 0.626; 10‐mo: *p* = 0.599). However, the immunofluorescence signals of Iba1 and the in vitro binding of [^11^C]Ac5216 were increased, and the immunofluorescence signals of P2Y12 receptor were decreased in the brainstem of PS19/p62‐KO mice compared with PS19 mice at 10 months of age (Figure 3f, *p* = 0.009, and Figure S6). The immunofluorescence signals of GFAP and C3 in the brainstem did not significantly differ between PS19/p62‐KO and PS19 (Figure 3g, and Figure S6). The immunofluorescence signals of AT8 and Iba1 were increased in the cortex of PS19/p62‐KO mice compared with PS19 mice at 10 months of age (Figure S7). p62 deficiency alone did not induce definite neuroinflammation in the hippocampus and brainstem of mice (Figure S8). These results suggest that abnormal tau species, which are suppressed by p62, cause cytotoxicity as well as consequent neuroinflammation and/or neuronal loss.

### 2.4 Enhanced accumulation of oligomeric tau species in p62‐deficient PS19 mice

To identify a certain tau species involved in neuronal loss and neuroinflammation, brain homogenates were fractionated by a sequential extraction method and examined for the recovery of tau protein in each fraction. Under physiological conditions, the majority of tau proteins were recovered in TBS. In a tauopathy mouse model, an intermediate form of tau aggregates can be extracted in a buffer with high salt and sucrose, and a late‐stage form of tau aggregates can be recovered in a detergent‐insoluble fraction (Sahara et al., 2013). The AT8‐positive tau contained in 1 M sucrose‐reassembly buffer (RAB) fraction was profoundly increased in the hippocampus of PS19/p62‐KO mice compared with PS19 mice, indicating a critical contribution of p62 to the processing of phosphorylated tau in this fraction (Figure S9; 8‐mo: *p* = 0.029, 10‐mo: *p* = 0.021). Although the AT8‐positive tau contained in the detergent‐insoluble FA fraction tended to increase in the hippocampus of PS19/p62‐KO mice compared with PS19 mice, the change was not significant (Figure S9). These results indicated that relatively immature tau aggregates (i.e., non‐filamentous) were major components of tau species accumulated in PS19/p62‐KO mice.

**FIGURE 4:**
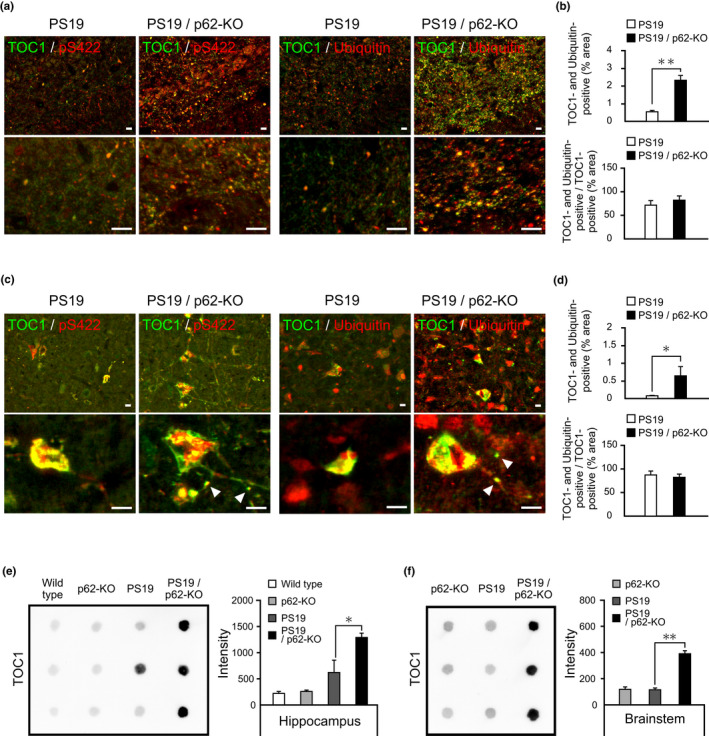
Accumulation of oligomeric tau species in PS19/p62‐KO mice. (a) Stratum lacunosum‐moleculare of hippocampus from 8‐month‐old PS19 and PS19/p62‐KO mice were immunostained with TOC1 (green) and pS422 (red) antibodies (left), or TOC1 (green) and anti‐ubiquitin (red) antibodies (right). Low (upper panels) and high (lower panels) magnification images are shown. (b) Positive areas of fluorescence staining were quantified. Double‐positive area of TOC1 and ubiquitin‐per‐unit area (%, upper) and double‐positive area of TOC1 and ubiquitin‐per‐TOC1‐positive area (%, lower) were compared between stratum lacunosum‐moleculare of PS19 and PS19/p62‐KO mice (*n* = 3, respectively). Data are presented as mean ± SD. **p < 0.005 by Welch's *t*‐test. (c) The brainstems from 10‐month‐old PS19 and PS19/p62‐KO mice were immunostained with TOC1 (green) and pS422 (red) antibodies (left), or TOC1 (green) and anti‐ubiquitin (red) antibodies (right). Low (upper panels) and high (lower panels) magnification images are shown. Diffuse double‐labeling (arrowheads) with TOC1 and pS422 antibodies or TOC1 and ubiquitin antibodies were observed in the brainstem of PS19/p62‐KO mice. (d) Positive areas of fluorescence staining were quantified. Double‐positive area of TOC1 and ubiquitin‐per‐unit area (%, upper) and double‐positive area of TOC1 and ubiquitin‐per‐TOC1‐positive area (%, lower) were compared between brainstem of PS19 and PS19/p62‐KO mice (*n* = 3, respectively). Data are presented as mean ± SD. *p < 0.05 by Welch's *t*‐test. (e) Representative dot‐blot image of hippocampal extracts labeled by TOC1 antibody. Equal volumes of 1 M sucrose‐RAB fractions from the hippocampi of 8‐month‐old wild‐type, p62‐KO, PS19, and PS19/p62‐KO mice were spotted on nitrocellulose membranes, and incubated with TOC1 antibody. Signals were detected by HRP‐conjugated anti‐IgM antibody and enhanced chemiluminescence method. Dot‐blot signals were quantified (*n* = 3, respectively). Data are presented as mean ± SEM. *p < 0.05 by one‐way ANOVA (group) followed by Tukey's HSD test. (f) Representative dot‐blot image of brainstem extracts labeled by TOC1 antibody. Equal volumes of 1 M sucrose‐RAB fractions from the brainstems of 10‐month‐old p62‐KO, PS19, and PS19/p62‐KO mice were spotted on nitrocellulose membranes and incubated with TOC1 antibody. Signals were detected by HRP‐conjugated anti‐IgM antibody and enhanced chemiluminescence method. Dot‐blot signals were quantified (*n* = 3, respectively). Data are presented as mean ± SEM. **p < 0.005 by one‐way ANOVA (group) followed by Tukey's HSD test. Scale bars, 20 μm

To further confirm the existence of immature aggregated tau species, immunostaining and dot‐blot analyses with tau oligomeric complex 1 (TOC1) antibody, which selectively recognize tau dimers and higher‐order oligomers over monomers or filamentous aggregates, were performed. Immunolabeling demonstrated that TOC1 signals were co‐localized with phospho‐Ser 422 tau immunoreactivity (an early pre‐tangle phosphoepitope in tau) in the hippocampus of PS19/p62‐KO mice at 8 months of age, but little co‐localization was observed in PS19 mice (Figure 4a). In addition, TOC1 signals that co‐localized with ubiquitin immunoreactivity were significantly increased in the stratum lacunosum‐moleculare of the hippocampus of PS19/p62‐KO mice compared to PS19 mice (Figure 4a,b). In the brainstem, robust co‐localization between TOC1 and pS422 antibodies or TOC1 and ubiquitin antibodies was observed in cell bodies of PS19 and PS19/p62‐KO mice at 10 months of age (Figure 4c). TOC1 signals that co‐localized with ubiquitin immunoreactivity were significantly increased in the brainstems of PS19/p62‐KO mice compared to PS19 mice (Figure 4c,d). In addition, a part of phosphorylated tau accumulated as granular dot‐like aggregates, as seen in the hippocampus of PS19/p62‐KO mice, taking an oligomeric form including ubiquitin in the brainstem of PS19/p62‐KO mice (Figure 4c, arrowheads). Moreover, dot‐blot analysis using TOC1 antibody illustrated age‐dependent accumulation of oligomeric tau in the TBS‐soluble fraction from the hippocampus of PS19 mice (data not shown) and significant increase in the 1 M sucrose‐RAB fraction of PS19/p62‐KO mice compared with PS19 mice at 8 months of age (Figure 4e; group *F*(3, 8) = 16.8, *p* = 0.01, PS19 vs PS19/p62‐KO: *p* = 0.019), although the concentrations of total human tau proteins were unaltered (Figure S10; group *F*(3, 8) = 11.851, *p* = 0.003, PS19 vs PS19/p62‐KO: *p* = 0.449). The accumulation of oligomeric tau in the brainstem of PS19/p62‐KO mice at 10 months of age was also confirmed by dot‐blot analysis (Figure 4f; group *F*(2, 6) = 105.5, *p* < 0.0001, PS19 vs PS19/p62‐KO: *p* < 0.0001). These data indicate that p62 deficiency accelerates the accumulation of oligomeric tau species in the PS19 mice. Immunolabeling also demonstrated the co‐localization of oligomeric tau, p62, and ubiquitin in the hippocampus of AD, striatum of PSP, frontal cortex of CBD, and frontal cortex of PiD patients (Figure S11).

### 2.5 Central role of p62 in the elimination of toxic tau species

**FIGURE 5:**
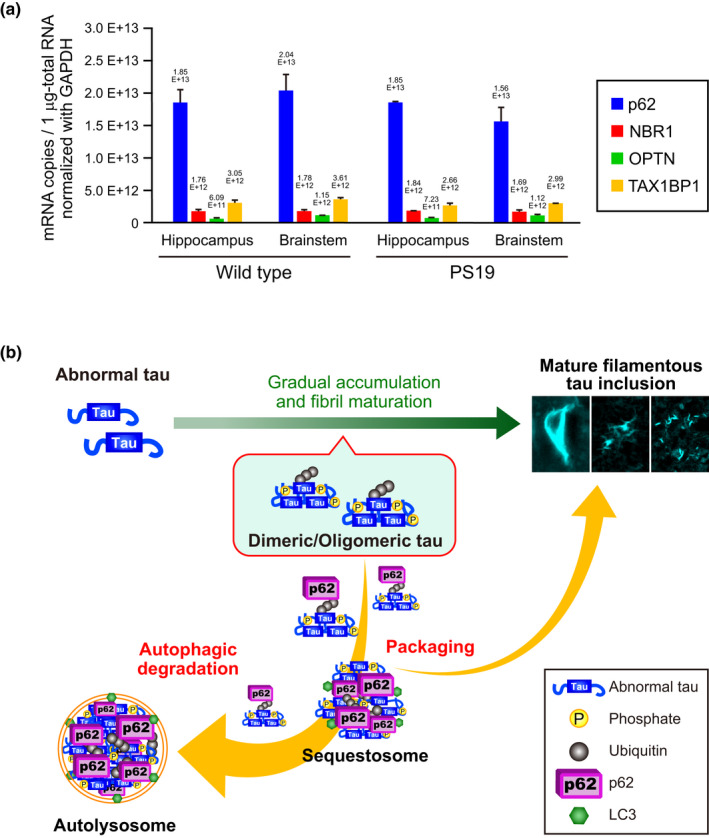
Pivotal roles of p62 in neuroprotection revealed with a tauopathy mouse model. (a)The absolute mRNA amounts of autophagy receptors in the hippocampus and brainstem of 11‐month‐old PS19 and wild‐type mice (*n* = 2, respectively) were calculated by quantitative RT‐PCR analysis. GFP‐fused autophagy receptor plasmids were used as quantification standards, and each plasmid amount was normalized to the copy number of the GFP gene. The mRNA copies of each autophagy receptor in 1 μg‐total RNA normalized with GAPDH are shown. Data are presented as mean ± SD. (b) Pivotal role of p62 in neuroprotection against neuronal death as tau pathology was suggested in this study. p62 exerts neuroprotection by eliminating disease‐related oligomeric tau, formed during the maturation of tau fibrils, primarily through selective autophagy. Moreover, disease‐related oligomeric tau may be packed by p62 into mature filamentous tau inclusions

Our data demonstrated that p62 prevents accumulation of oligomeric tau species. The mechanism of this prevention is considered to be via the elimination of oligomeric tau species by p62. However, a previous study demonstrated that mice deficient in the p62 gene develop mature‐onset obesity and insulin resistance (Bhat & Thirumangalakudi, 2013). Defective central insulin signaling secondary to obesity‐related peripheral insulin resistance may induce tau phosphorylation via activation of GSK‐3β (Bhat & Thirumangalakudi, 2013). Therefore, we analyzed the activities of the tau kinases GSK‐3β and CDK5 in PS19/p62‐KO mice. Deficiency of p62 in PS19 mice did not affect the activities of the tau kinases GSK‐3β (Figure S12a,c; GSK‐3β, brainstem: *p* = 0.990, hippocampus: *p* = 0.574; P‐GSK‐3β, brainstem: *p* = 0.395, hippocampus: *p* = 0.913) and Cdk5 (Figure S12b,c; p25/p35, brainstem: *p* = 0.207, hippocampus: *p* = 0.301; CDK5, brainstem: *p* = 0.063, hippocampus: *p* = 0.885). Therefore, the increase of oligomeric tau species observed in the hippocampus and brainstem of PS19/p62‐KO mice does not originate from an increasing activity of some known tau kinases, and it is rather assumed to be due to the absence of elimination of oligomeric tau species by p62 primarily through selective autophagy. We also measured the mRNA copies of each autophagy receptor—p62, NBR1, OPTN, and TAX1BP1—in the hippocampus and brainstem of 11‐month‐old wild‐type and PS19 mice by quantitative RT‐PCR (Figure 5a). The p62 mRNA level was more than fivefold higher than that of other ubiquitin‐dependent autophagy receptors. These results suggested that p62 is the most abundant and major autophagy receptor utilized for elimination of ubiquitinated protein under proteotoxic stress conditions in the hippocampus and brainstem of wild‐type and PS19 mice.

## 3 DISCUSSION

In this study, we established that the removal of p62 exacerbates tau pathologies, neuronal death, and neuroinflammation in a mouse model of tauopathy. Genetic inactivation of p62 is thought to cause an accumulation of abnormally phosphorylated tau aggregates (Babu et al., 2008). We also demonstrated an association between p62 and specific pathogenic species of tau, for example, tau oligomers. The knocking out of p62 in PS19 mice caused significant accumulation of tau oligomers. Since tau oligomers are disease‐specific and are likely involved in the neurotoxicity and pathogenesis of tauopathies (Lasagna‐Reeves et al., 2010; Patterson et al., 2011), our findings demonstrated a key role for p62 in the modulating accumulation of tau oligomers and the neurodegeneration associated with these forms of tau.

In recent years, a great body of evidence has suggested that NFTs are not the primary toxic tau aggregate within the brain in AD and other tauopathies (Santacruz et al., 2005). Alternatively, it has been proposed that tau oligomers, a pre‐filament form of tau, are responsible for a large part of disease‐related neurotoxicity (Berger et al., 2007; Maeda et al., 2007). When tau monomers, oligomers, and fibrils were injected into the CA1 region of the hippocampus in wild‐type mice, tau oligomers caused memory deficits and cell damage, whereas tau fibrils and monomers did not (Lasagna‐Reeves et al., 2011). In the current study, we provided the first evidence that accumulation of tau oligomers is increased in a mouse model of tauopathy by the genetic elimination of p62, in concurrence with neuron death. Our data support the view that oligomeric tau is an acutely toxic tau species and induces neurodegeneration. Although various studies have demonstrated the association between macroautophagy and abnormally phosphorylated tau accumulation (Ozcelik et al., 2013; Schaeffer et al., 2012), the underlying mechanism of this modulation has been unclear. Our data indicate that p62 preferentially prevents accumulation of oligomeric tau in PS19 brain without affecting the level of total tau protein, late‐stage form of tau aggregates, and activities of typical tau kinases. These observations imply that p62 eliminates oligomeric tau species primarily through selective autophagy, and future efforts to analyze the direct interaction between p62 and ubiquitinated tau oligomers may be necessary to gain more insight. Furthermore, identifying which polyubiquitin chain, K48‐linked or K‐63‐linked, is added to tau oligomers will be important for clarifying the polyubiquitination of abnormal tau.

A previous study investigated the distinct roles of cargo receptors, such as OPTN and p62, in which PTEN‐induced kinase 1 (PINK1)‐dependent mitophagy required OPTN but not p62 (Matsumoto et al., 2015). More recently, Xu et al. (2019) demonstrated that OPTN facilitated normal soluble tau degradation while p62 primarily targeted misfolded and insoluble tau for selective autophagy. In P301L tau transgenic mice, p62 overexpression by somatic transgenic gene delivery prevented accumulation of insoluble hyperphosphorylated tau without reducing soluble tau species (Xu et al., 2019). Those results confirmed the p62 function targeting insoluble mutant tau species leading to the reduction of tau pathology. Although these upregulating paradigms were not examined in the present study, the physiological expression of p62 in PS19 mice protects against massive neuronal loss and tau pathology, which were expanded in PS19/p62‐KO mice. As p62 binds to polyubiquitinated proteins and generates cytoplasmic structure called sequestosome, which is defined as the cytoplasmic punctate structure of p62 without membrane confinements (Bjorkoy et al., 2005; Shin, 1998), entrapment of ubiquitinated tau into sequestosome by p62 followed by autophagic degradation may be beneficial for neuronal protection. Recent studies also demonstrated that ubiquitinated protein‐bound p62 forms liquid droplet as sequestosome by liquid–liquid phase separation (Kageyama et al., 2021; Zaffagnini et al., 2018), and phosphorylation of p62 at S403 stabilizes the sequestosome structure and enhances the selective autophagy of ubiquitinated protein (Matsumoto et al., 2011). Since several studies have reported the favorable effects of autophagic activation, in a manner upregulating autophagosome formation, on tau pathology (Congdon et al., 2012; Ozcelik et al., 2013; Schaeffer et al., 2012), simultaneous upregulation of p62 may be a promising treatment for the amelioration of tau pathology.

p62‐KO mice develop mature‐onset obesity associated with leptin resistance (Harada et al., 2013; Rodriguez et al., 2006). Nevertheless, suppression of weight gain occurred when crossbreeding p62‐KO mice with PS19 mice in the present study (Figure S12d). With the suppression of weight gain, elevations of serum insulin and blood glucose levels were not observed in PS19/p62‐KO mice (Figure S12e,f). In PS19 mice, mature‐onset weight loss associated with limb paralysis was observed (Merchán‐Rubira et al., 2019). Part of the suppression of weight gain in PS19/p62‐KO mice is caused by limb paralysis, which was observed in old PS19/p62‐KO mice but not in littermate p62‐KO mice. However, we assume that the suppression of weight gain is mainly provoked by a decrease in leptin resistance, which resulted from crossbreeding with PS19 mice. Statistical analysis showed no significant difference in serum leptin level between PS19/p62‐KO and PS19 or wild‐type mice, although the serum leptin level was significantly increased in p62‐KO mice in comparison with PS19 and wild‐type mice (Figure S12g). In p62‐KO mice, leptin resistance is considered to be caused by a defect in the intracellular distribution of transcription factor Stat3 provoked by a lack of p62 (Harada et al., 2013). Although the effects of the central leptin‐signaling pathway including PI3K/Akt and JAK/STAT on tau phosphorylation were demonstrated in previous studies (Cross et al., 1995; Huang et al., 2007), the effect of abnormal tau accumulation on the central leptin‐signaling pathway is still unclear. Analysis of Stat3 and other factors involved in the leptin‐signaling pathway in the brains of PS19/p62‐KO mice may provide new knowledge concerning the leptin‐signaling pathway. Furthermore, earlier decrease of estrogen levels with aging is suggested in p62‐KO female mice compared with wild‐type female mice (Ishii T. and Warabi E., unpublished observation). Previous studies demonstrated that estrogen suppresses tau phosphorylation by downregulation/upregulation of the activity of protein kinase such as GSK‐3β and Cdk5, and protein phosphatase (Goodenough et al., 2005; Liu et al., 2008; Shi et al., 2008). Since there is no significant difference in the levels of GSK‐3β, phospho‐Ser9 GSK‐3β, Cdk5, p25, and p35 between PS19/p62‐KO and PS19 brains at 8 months of age, an increase of phosphorylated tau caused by p62 deficiency in PS19 mice is unlikely due to activity changes of these kinases caused by reduction of the estrogen level. Further studies of estrogen‐induced kinase activation will be necessary to clarify the contribution of estrogen to the tau pathology in PS19/p62‐KO female mice.

Our study demonstrated that accumulation of phosphorylated tau and formation of NFT‐like inclusions occurred in neuronal somas in the brainstem of PS19/p62‐KO mice (Figure 4b and Figure S13), suggesting that the function of p62 in packaging the ubiquitinated proteins into inclusion bodies (Moscat et al., 2007) is not essential for NFT formation. On the contrary, our findings also support the notion that p62 may partially contribute to the packaging of oligomeric tau into filamentous tau inclusions, as we determined that oligomeric tau species including ubiquitin accumulated as granular dot‐like aggregates in the area outside neuronal somas in the brainstem of PS19/p62‐KO mice (Figure 4b). Therefore, we propose a mechanism that p62 acts protectively against neuronal death and neuroinflammation by eliminating and partially packaging disease‐related oligomeric tau formed during the maturation of tau fibrils (Figure 5b).

## 4 EXPERIMENTAL PROCEDURES

### 4.1 Mice

P301S mutant human tau transgenic mice with C57BL/6 background (line PS19) were generated by using a cDNA construct of human T34 tau with the P301S mutation in combination with a murine prion protein promoter (Yoshiyama et al., 2007). p62‐knock‐out (KO) mice (*p62* ^−/−^) with a C57BL/6 background were generated by using a targeting vector designed for the deletion of exons 1–4 of the mouse *p62*/*A170* gene as described previously (Komatsu et al., 2007). Double‐mutant PS19/p62‐KO mice (*Tau ^P301S^* ^/+^:*p62* ^−/−^) were generated by breeding p62‐KO mice with PS19 mice. Female mice were used for the experiments. All experimental procedures related to animals and their care were approved by the Animal Ethics Committee of the National Institutes for Quantum Science and Technology.

### 4.2 Antibodies and compounds

The following antibodies were used: mouse monoclonal antibody against tau protein phosphorylated at Ser202 and Thr205 (AT8, MN1020, Thermo Fisher Scientific, USA), tau protein phosphorylated at Ser396 and Ser404 (PHF1, provided by Dr. P. Davies), an epitope near the C‐terminal region (404–441 aa) of human tau protein (T46, 13–6400, Thermo Fisher Scientific; Tau 12, provided by Dr. N. M. Kanaan), tau oligomers (TOC1, provided by Dr. N. M. Kanaan) (Patterson et al., 2011; Ward et al., 2014), β‐actin (A1978, Merck, Germany), and NeuN (MAB377, Merck), rat monoclonal antibody against glial fibrillary acidic protein (GFAP, 13–0300, Thermo Fisher Scientific) and complement C3 (NB200‐540, Novus, USA), guinea big polyclonal antibody against human p62 protein (GP62‐C, PROGEN, Germany), rabbit polyclonal antibody against human p62 protein (PM045, MBL, Japan), human tau protein (E1, provided by Dr. S.‐H. Yen), phosphorylated tau protein at Ser422 (pS422, 44‐764G, Thermo Fisher Scientific), ubiquitin (Z0458, DAKO, Denmark), ionized calcium‐binding adapter molecule‐1 (Iba1, 019‐19741, Wako, Japan), mouse P2Y12 receptor (Maeda et al., 2021), glycogen synthase kinase 3β (GSK‐3β, 5676S, Cell Signaling Technology, USA), phosphorylated GSK‐3β at Ser9 (P‐GSK‐3β, 9336S, Cell Signaling Technology), p25 and p35 (2680S, Cell Signaling Technology), and CDK5 (2506S, Cell Signaling Technology). The β‐sheet‐binding compound FSB, (E, E)‐1‐fluoro‐2, 5‐bis (3‐hydroxycarbonyl‐4‐hydroxy) styrylbenzene, is commercially available (B525, Dojindo, Japan).

### 4.3 Histological examination

Mice were intraperitoneally anesthetized with 20 mg/kg sodium pentobarbital (Somnopentyl, Kyoritsu Seiyaku, Japan). Brains were removed after perfusion with phosphate‐buffered saline (PBS) and fixed with 4% paraformaldehyde in PBS. After cryoprotection using 30% sucrose in PBS, 20‐μm‐thick frozen coronal sections were prepared by cryostat (HM560; Thermo Fisher Scientific).

All brain autopsies were performed after consent from a legal next of kin or from an individual with power of attorney. Postmortem human brains were obtained from autopsies carried out at the Center for Neurodegenerative Disease Research of the University of Pennsylvania Perelman School of Medicine on patients with AD and non‐AD tauopathy and healthy control subjects. Studies of autopsy samples were approved by the National Institutes for Quantum Science and Technology Certified Review Board (approval number: 14‐015). Tissues were fixed in 10% formalin and embedded in paraffin blocks. 6‐μm‐thick sections were prepared with a microtome (SM2000R, LEICA Microsystems, Germany).

Sections were autoclaved in citrate buffer for antigen retrieval except when using Iba1, GFAP, TOC1, and a subset of p62 antibodies. Immunostaining was performed by standard protocol using fluorophore‐conjugated secondary antibodies, HRP labeled polymer conjugated with secondary antibodies, a commercial kit for Tyramide Signal Amplification (TSA system, Perkin Elmer, USA), and a kit for avidin‐biotin‐diaminobenzidine staining (ABC Staining Systems, Santa Cruz, USA), respectively. For fluorescence staining, brain sections were incubated with 0.01% FSB in 50% ethanol for 30 min at 25°C. The sections were quickly differentiated in saturated Li_2_CO_3_ and washed with 50% ethanol. Stained sections were analyzed by DM4000 microscope (LEICA Microsystems), BX51 microscope (Olympus, Japan), BZ‐9000 microscope (KEYENCE, Japan), BZ‐X710 microscope (KEYENCE), and LSM 880 (Zeiss, Germany). Signal intensities and areas of immunofluorescence staining were quantified using Metamorph (Molecular Devices, Canada) and BZ‐X Analyser (KEYENCE).

### 4.4 MRI experiments

PS19/p62‐KO and PS19 mice (female, 8 and 10 months old, respectively) were scanned by 7.0‐T MRI scanner (Magnet: Kobelco and JASTEC, Japan; Console: Bruker Biospin, Germany) with a volume coil for transmission (Bruker) and a two‐channel phased‐array coil for reception (Rapid Biomedical, Germany). Signal excitation and detection were performed using a 25‐mm resonator. Mice were anesthetized with isoflurane, and T2‐weighted 3D spin‐echo MR images were acquired by rapid acquisition with relaxation enhancement (RARE) sequence (field of view = 25.6 × 14.4 × 16.0, matrix dimensions = 256 × 256 × 32, nominal resolution = 0.1 × 0.056 × 0.5, effective echo time = 36 msec, repetition time = 4200 msec, RARE factor = 8, number of acquisitions = 8, and total imaging time = 14 min 26 s). Specific emphasis was placed on the hippocampus and brainstem in coronal images (1.0–4.0 and 5.0–8.5 mm posterior to the bregma), and regional volumes were determined using PMOD software (PMOD Technologies, Switzerland).

### 4.5 Tissue extraction and immunoblot analyses

Frozen brain tissues were homogenized in TBS (50 mM Tris/HCl [pH 7.4], 274 mM NaCl, 5 mM KCl, 1% protease inhibitor mixture (1% phosphatase inhibitor cocktail I & II (Thermo Fisher Scientific)), 1 mM phenylmethylsulfonyl fluoride [PMSF]). The protein amounts of the homogenates were determined by Lowry protein assay, and samples were diluted to 5‐mg protein in 1 ml of homogenate buffer. These brain homogenates and a standard homogenate (as a loading control) prepared from the brain of PS19 mice were applied to SDS‐PAGE and separated proteins were transferred onto a polyvinylidene fluoride (PVDF) membrane (Immobilon‐P, Merck). After blocking with a blocking solution containing 0.1% BSA and 0.05% Tween 20 in TBS, the membranes were incubated with primary antibodies, washed to remove excess antibodies, and then incubated with HRP‐conjugated anti‐IgG antibodies. Bound antibodies were detected using an enhanced chemiluminescence system (GE Healthcare, UK). Western blot immunoreactivity was visualized by computer‐linked ChemiDoc XRS image analyzer (Bio‐Rad Laboratories, USA), and quantitative analysis was performed by Quantity One software (Bio‐Rad Laboratories). The results were standardized across trials using the signal of a loading control sample.

To obtain buffer‐soluble and sarkosyl‐insoluble fractions, brain regions were homogenized in 10 volumes of TBS. The homogenates were centrifuged at 27,000 *g* for 20 min at 4°C to obtain supernatant (TBS soluble) and pellet fractions. Pellets were further homogenized in five volumes for brains of high salt/sucrose buffer (0.8 M NaCl, 10% sucrose, 10 mM Tris/HCl, [pH 7.4], 1 mM EGTA, and 1 mM PMSF) and centrifuged as above. Supernatants were collected and incubated with sarkosyl (1% final concentration, Thermo Fisher Scientific) for 1 h at 37°C, followed by centrifugation at 150,000 *g* for 1 h at 4°C to obtain salt and sarkosyl‐extractable and sarkosyl‐insoluble fractions. The sarkosyl‐insoluble pellet was resuspended in TE buffer (10 mM Tris/HCl (pH 8.0), 1 mM EDTA) to a volume equivalent to half of that of the brain specimens used to produce brain homogenates.

To determine tau protein solubility, sequential extraction with different buffer contents was performed according to previous reports (Kraemer et al., 2003; Tanemura et al., 2002). Brain homogenates were centrifuged at 50,000 *g* for 40 min at 4°C. Supernatants were recovered as a TBS fraction. The pellets were resuspended with RAB (0.1 M MES, 1 mM EGTA, 0.5 mM MgSO_4_, 0.75 M NaCl, 0.02 M NaF, 1 mM PMSF, and 0.1% protease inhibitor mixture) containing 1 M sucrose and centrifuged at 50,000 *g* for 20 min at 4°C. Supernatants were recovered as a 1 M sucrose‐RAB fraction. The pellets were resuspended with radioimmunoprecipitation assay (RIPA) buffer (50 mM Tris‐HCl, 150 mM NaCl, 0.5% sodium deoxycholate, 0.1% SDS, 1% NP‐40, 0.5 mM PMSF, pH8.0). The RIPA fraction was recovered in the supernatants from centrifugation at 100,000 *g* for 20 min at 4°C. The resultant pellets were suspended with 70% formic acid (FA) and collected as FA fraction. 1 M sucrose‐RAB, RIPA, and FA fractions were applied to SDS‐PAGE, and proteins were transferred onto PVDF membranes. Phosphorylated tau proteins were detected by AT8 antibody, and the distribution of AT8‐positive tau in each fraction was determined by quantitative analysis. In parallel, dot‐blot analysis for detecting oligomeric tau species was performed by TOC1 antibody according to previous reports (Sahara et al., 2013; Ward et al., 2014). Briefly, 2 μl of 1 M sucrose‐RAB fractions was spotted onto a nitrocellulose membrane (Bio‐Rad Laboratories) and then blocked by blocking solution containing 5% non‐fat dry milk and 0.05% Tween 20 in TBS. After overnight incubation with primary antibodies, membranes were reacted with HRP‐conjugated goat anti‐mouse IgG or IgM antibody (Jackson ImmunoResearch, USA). Bound antibodies were detected by an enhanced chemiluminescence system. The signals were measured by image quantification software (Quantity One, Bio‐Rad Laboratories).

### 4.6 Measurements for blood glucose, serum leptin, and serum insulin

After a 16‐h overnight fast, the blood glucose level was measured by glucose meter (NIPRO, Japan). Body weights were also measured, and blood samples were obtained via tails, followed by centrifugation at 800 *g* for 10 min. Blood serums were transferred and used in ELISA assay kits for measurement of serum leptin and insulin levels (leptin, IBL, Japan; insulin, Mercodia AB, Sweden). Optical densities were read at 450 nm by Safire II Plate Reader (Tecan, Switzerland).

### 4.7 Quantitative real‐time PCR

cDNA was synthesized from 1 μg of total RNA using the Transcriptor First Strand cDNA Synthesis Kit (Roche, Switzerland). Quantitative PCR was performed with LightCycler 480 Probe Master (Roche). Signals were normalized to b‐glucuronidase. The sequences of primers used were p62F: 5′‐TGTGGAACATGGAGGGAAGAG‐3′; p62R: 5′‐TGTGCCTGTGCTGGAACTTTC‐3′; and p62P: 5′‐AGCCGCCTGACACCCACTACCCC‐3′, according to those previously reported (Komatsu et al., 2010). Quantitative PCR analysis was also performed using LightCycler 480 (Roche) and THUNDERBIRD^®^ NextSYBR^®^ qPCR Mix (TOYOBO, Japan) according to the manufacturer's protocol. Primer sets have been previously reported (Matsumoto et al., 2018) and are designed using the Roche Universal Probe Library Assay Design Center program (Roche) based on the following accession numbers: mp62/SQSTM1/A170: NM_011018, mNBR1: NM_008676, mOPTN: NM_181848, mTAX1BP1: NM_025816, mGapdh: NM_008084. Sequence information was also used to clone each gene. Gapdh was used for normalization. A GFP‐fused autophagy receptor plasmid was used to quantify the absolute amount of autophagy receptor. In this plasmid, the open reading frame sequence of each mouse autophagy receptor was cloned into the pEGFP‐C1 plasmid (clontech). The PCR amplification standard curve for each autophagy receptor was created using a serially diluted plasmid, and the standard curve was converted into the number of copies of the plasmid DNA containing each autophagy receptor gene. The number of copies of plasmid DNA was further normalized with the GFP gene. We calculated the absolute amount of mRNA in the same mRNA pool and compared the numbers of copies of each mRNA. Whole RNA pools were prepared from the hippocampus and brainstem of two littermate PS19 wild and transgenic mice using TRI Reagent^®^ (Molecular Research Center, USA). The entire RNA pool was reverse transcribed using PrimeScript ™ RT Master Mix (Takara Bio, Japan) to generate cDNA.

### 4.8 Radiosynthesis and autoradiography

[^11^C]Ac5216, a PET ligand for translocator protein (TSPO), was synthesized as described previously (Zhang et al., 2007). In brief, Ac5216 and its desmethyl precursor were synthesized starting from commercially available compounds. [^11^C]Ac5216 was radiosynthesized through the reaction of the precursor with [^11^C]CH_3_I in the presence of sodium hydride. In vitro autoradiography of TSPO was performed using fixed brain sections. The samples were incubated with [^11^C]Ac5216 (125.8 M Bq/L, 1 nM) in 50 mM Tris‐HCl. Nonspecific binding of the radioligand was estimated by adding nonradioactive PK11195 (10 μM) to the reaction. The samples were then rinsed twice with ice‐cold Tris‐HCl buffer for 2 min, dipped into ice‐cold water for 10 s, warmly blow‐dried, and contacted to an imaging plate (BAS‐MS2025; Fuji Film, Japan) for 1 h. Radiolabeling was detected by scanning the imaging plate by means of the BAS5000 system (Fuji Film). Signal intensities of autoradiography were quantified using Multi Gauge (Fuji Film).

### 4.9 Statistical analyses

Statistical significance of the data was analyzed with GraphPad Prism software (Graphpad Inc., USA). Values of *p* < 0.05 indicated statistical significance. For all graphs, the group sizes and statistical tests used are indicated in each figure legend.

## CONFLICT OF INTEREST

The authors declare that they have no competing interests.

## AUTHOR CONTRIBUTIONS

MO, NS, and MH conceived and designed the project. MO, MK, BJ, YT, MS, and TM performed the experimental work. EW, TY, IA, and NMK provided the resources. TS, NS, and MH directed the work. All authors reviewed and edited the manuscript.

## Supporting information

Supplementary Material

## Data Availability

The datasets used and/or analyzed during the current study are available from the corresponding authors, Maiko Ono and Naruhiko Sahara, upon reasonable request.

## References

[acel13615-bib-0001] Arriagada, P. V. , Growdon, J. H. , Hedley‐Whyte, E. T. , & Hyman, B. T. (1992). Neurofibrillary tangles but not senile plaques parallel duration and severity of Alzheimer's disease. Neurology, 42, 631–639. 10.1212/wnl.42.3.631 1549228

[acel13615-bib-0002] Babu, J. R. , Seibenhener, M. L. , Peng, J. , Strom, A.‐L. , Kemppainen, R. , Cox, N. , Zhu, H. , Wooten, M. C. , Diaz‐Meco, M. T. , Moscat, J. , & Wooten, M. W. (2008). Genetic inactivation of p62 leads to accumulation of hyperphosphorylated tau and neurodegeneration. Journal of Neurochemistry, 106, 107–120. 10.1111/j.1471-4159.2008.05340.x 18346206

[acel13615-bib-0003] Berger, Z. , Roder, H. , Hanna, A. , Carlson, A. , Rangachari, V. , Yue, M. , Wszolek, Z. , Ashe, K. , Knight, J. , Dickson, D. , Andorfer, C. , Rosenberry, T. L. , Lewis, J. , Hutton, M. , & Janus, C. (2007). Accumulation of pathological tau species and memory loss in a conditional model of tauopathy. Journal of Neuroscience, 27, 3650–3662. 10.1523/JNEUROSCI.0587-07.2007 17409229 PMC6672413

[acel13615-bib-0004] Bhat, N. R. , & Thirumangalakudi, L. (2013). Increased tau phosphorylation and impaired brain insulin/IGF signaling in mice fed a high fat/high cholesterol diet. Journal of Alzheimer's Disease, 36, 781–789. 10.3233/JAD-2012-121030 PMC4445975 23703152

[acel13615-bib-0005] Bjorkoy, G. , Lamark, T. , Brech, A. , Outzen, H. , Perander, M. , Overvatn, A. , Stenmark, H. , & Johansen, T. (2005). p62/SQSTM1 forms protein aggregates degraded by autophagy and has a protective effect on huntingtin‐induced cell death. Journal of Cell Biology, 171, 603–614. 10.1083/jcb.200507002 16286508 PMC2171557

[acel13615-bib-0006] Congdon, E. E. , Wu, J. W. , Myeku, N. , Figueroa, Y. H. , Herman, M. , Marinec, P. S. , Gestwicki, J. E. , Dickey, C. A. , Yu, W. H. , & Duff, K. E. (2012). Methylthioninium chloride (methylene blue) induces autophagy and attenuates tauopathy in vitro and in vivo. Autophagy, 8, 609–622. 10.4161/auto.19048 22361619 PMC3405840

[acel13615-bib-0007] Cross, D. A. , Alessi, D. R. , Cohen, P. , Andjelkovich, M. , & Hemmings, B. A. (1995). Inhibition of glycogen synthase kinase‐3 by insulin mediated by protein kinase B. Nature, 378, 785–789. 10.1038/378785a0 8524413

[acel13615-bib-0008] Goodenough, S. , Schleusner, D. , Pietrzik, C. , Skutella, T. , & Behl, C. (2005). Glycogen synthase kinase 3beta links neuroprotection by 17beta‐estradiol to key Alzheimer processes. Neuroscience, 132, 581–589. 10.1016/j.neuroscience.2004.12.029 15837120

[acel13615-bib-0009] Harada, H. , Warabi, E. , Matsuki, T. , Yanagawa, T. , Okada, K. , Uwayama, J. , Ikeda, A. , Nakaso, K. , Kirii, K. , Noguchi, N. , Bukawa, H. , Siow, R. C. M. , Mann, G. E. , Shoda, J. , Ishii, T. , & Sakurai, T. (2013). Deficiency of p62/Sequestosome 1 causes hyperphagia due to leptin resistance in the brain. Journal of Neuroscience, 33, 14767–14777. 10.1523/JNEUROSCI.2954-12.2013 24027277 PMC6705174

[acel13615-bib-0010] Huang, F. , Kao, C. Y. , Wachi, S. , Thai, P. , Ryu, J. , & Wu, R. (2007). Requirement for both JAK‐mediated PI3K signaling and ACT1/TRAF6/TAK1‐dependent NF‐kappaB activation by IL‐17A in enhancing cytokine expression in human airway epithelial cells. The Journal of Immunology, 179, 6504–6513. 10.4049/jimmunol.179.10.6504 17982039

[acel13615-bib-0011] Jadhav, S. , Avila, J. , Scholl, M. , Kovacs, G. G. , Kovari, E. , Skrabana, R. , Evans, L. D. , Kontsekova, E. , Malawska, B. , de Silva, R. , Buee, L. , & Zilka, N. (2019). A walk through tau therapeutic strategies. Acta Neuropathologica Communications, 7, 22. 10.1186/s40478-019-0664-z 30767766 PMC6376692

[acel13615-bib-0012] Jankowsky, J. L. , & Zheng, H. (2017). Practical considerations for choosing a mouse model of Alzheimer's disease. Molecular Neurodegeneration, 12, 89. 10.1186/s13024-017-0231-7 29273078 PMC5741956

[acel13615-bib-0013] Kageyama, S. , Gudmundsson, S. R. , Sou, Y.‐S. , Ichimura, Y. , Tamura, N. , Kazuno, S. , Ueno, T. , Miura, Y. , Noshiro, D. , Abe, M. , Mizushima, T. , Miura, N. , Okuda, S. , Motohashi, H. , Lee, J.‐A. , Sakimura, K. , Ohe, T. , Noda, N. N. , Waguri, S. , … Komatsu, M. (2021). p62/SQSTM1‐droplet serves as a platform for autophagosome formation and anti‐oxidative stress response. Nature Communications, 12, 16. 10.1038/s41467-020-20185-1 PMC7782522 33397898

[acel13615-bib-0014] Katsuragi, Y. , Ichimura, Y. , & Komatsu, M. (2015). p62/SQSTM1 functions as a signaling hub and an autophagy adaptor. FEBS Journal, 282, 4672–4678. 10.1111/febs.13540 26432171

[acel13615-bib-0015] Komatsu, M. , Kurokawa, H. , Waguri, S. , Taguchi, K. , Kobayashi, A. , Ichimura, Y. , Sou, Y. S. , Ueno, I. , Sakamoto, A. , Tong, K. I. , Kim, M. , Nishito, Y. , Iemura, S. , Natsume, T. , Ueno, T. , Kominami, E. , Motohashi, H. , Tanaka, K. , & Yamamoto, M. (2010). The selective autophagy substrate p62 activates the stress responsive transcription factor Nrf2 through inactivation of Keap1. Nature Cell Biology, 12, 213–223. 10.1038/ncb2021 20173742

[acel13615-bib-0016] Komatsu, M. , Waguri, S. , Koike, M. , Sou, Y. S. , Ueno, T. , Hara, T. , Mizushima, N. , Iwata, J. , Ezaki, J. , Murata, S. , Hamazaki, J. , Nishito, Y. , Iemura, S. , Natsume, T. , Yanagawa, T. , Uwayama, J. , Warabi, E. , Yoshida, H. , Ishii, T. , … Tanaka, K. (2007). Homeostatic levels of p62 control cytoplasmic inclusion body formation in autophagy‐deficient mice. Cell, 131, 1149–1163. 10.1016/j.cell.2007.10.035 18083104

[acel13615-bib-0017] Kraemer, B. C. , Zhang, B. , Leverenz, J. B. , Thomas, J. H. , Trojanowski, J. Q. , & Schellenberg, G. D. (2003). Neurodegeneration and defective neurotransmission in a *Caenorhabditis elegans* model of tauopathy. Proceedings of the National Academy of Sciences of the United States of America, 100, 9980–9985. 10.1073/pnas.1533448100 12872001 PMC187908

[acel13615-bib-0018] Lasagna‐Reeves, C. A. , Castillo‐Carranza, D. L. , Guerrero‐Muoz, M. J. , Jackson, G. R. , & Kayed, R. (2010). Preparation and characterization of neurotoxic tau oligomers. Biochemistry, 49, 10039–10041. 10.1021/bi1016233 21047142

[acel13615-bib-0019] Lasagna‐Reeves, C. A. , Castillo‐Carranza, D. L. , Sengupta, U. , Clos, A. L. , Jackson, G. R. , & Kayed, R. (2011). Tau oligomers impair memory and induce synaptic and mitochondrial dysfunction in wild‐type mice. Molecular Neurodegeneration, 6, 39. 10.1186/1750-1326-6-39 21645391 PMC3224595

[acel13615-bib-0020] Lee, V. M. , Goedert, M. , & Trojanowski, J. Q. (2001). Neurodegenerative tauopathies. Annual Review of Neuroscience, 24, 1121–1159. 10.1146/annurev.neuro.24.1.1121 11520930

[acel13615-bib-0021] Leyns, C. E. G. , & Holtzman, D. M. (2017). Glial contributions to neurodegeneration in tauopathies. Molecular Neurodegeneration, 12, 50. 10.1186/s13024-017-0192-x 28662669 PMC5492997

[acel13615-bib-0022] Liu, X. A. , Zhu, L. Q. , Zhang, Q. , Shi, H. R. , Wang, S. H. , Wang, Q. , & Wang, J. Z. (2008). Estradiol attenuates tau hyperphosphorylation induced by upregulation of protein kinase‐A. Neurochemical Research, 33, 1811–1820. 10.1007/s11064-008-9638-4 18338250

[acel13615-bib-0023] Maeda, J. , Minamihisamatsu, T. , Shimojo, M. , Zhou, X. , Ono, M. , Matsuba, Y. , Ji, B. , Ishii, H. , Ogawa, M. , Akatsu, H. , Kaneda, D. , Hashizume, Y. , Robinson, J. L. , Lee, V. M. Y. , Saito, T. , Saido, T. C. , Trojanowski, J. Q. , Zhang, M. R. , Suhara, T. , … Sahara, N. (2021). Distinct microglial response against Alzheimer’s amyloid and tau pathologies characterized by P2Y12 receptor. Brain Communications, 3, fcab011. 10.1093/braincomms/fcab011 33644757 PMC7901060

[acel13615-bib-0024] Maeda, S. , Sahara, N. , Saito, Y. , Murayama, M. , Yoshiike, Y. , Kim, H. , Miyasaka, T. , Murayama, S. , Ikai, A. , & Takashima, A. (2007). Granular tau oligomers as intermediates of tau filaments. Biochemistry, 46, 3856–3861. 10.1021/bi061359o 17338548

[acel13615-bib-0025] Matsumoto, G. , Inobe, T. , Amano, T. , Murai, K. , Nukina, N. , & Mori, N. (2018). N‐Acyldopamine induces aggresome formation without proteasome inhibition and enhances protein aggregation via p62/SQSTM1 expression. Scientific Reports, 8, 9585. 10.1038/s41598-018-27872-6 29941919 PMC6018635

[acel13615-bib-0026] Matsumoto, G. , Shimogori, T. , Hattori, N. , & Nukina, N. (2015). TBK1 controls autophagosomal engulfment of polyubiquitinated mitochondria through p62/SQSTM1 phosphorylation. Human Molecular Genetics, 24, 4429–4442. 10.1093/hmg/ddv179 25972374

[acel13615-bib-0027] Matsumoto, G. , Wada, K. , Okuno, M. , Kurosawa, M. , & Nukina, N. (2011). Serine 403 phosphorylation of p62/SQSTM1 regulates selective autophagic clearance of ubiquitinated proteins. Molecular Cell, 44, 279–289. 10.1016/j.molcel.2011.07.039 22017874

[acel13615-bib-0028] Merchán‐Rubira, J. , Sebastián‐Serrano, Á. , Díaz‐Hernández, M. , Avila, J. , & Hernández, F. (2019). Peripheral nervous system effects in the PS19 tau transgenic mouse model of tauopathy. Neuroscience Letters, 698, 204–208. 10.1016/j.neulet.2019.01.031 30677432

[acel13615-bib-0029] Mori, H. , Kondo, J. , & Ihara, Y. (1987). Ubiquitin is a component of paired helical filaments in Alzheimer's disease. Science, 235, 1641–1644. 10.1126/science.3029875 3029875

[acel13615-bib-0030] Moscat, J. , Diaz‐Meco, M. T. , & Wooten, M. W. (2007). Signal integration and diversification through the p62 scaffold protein. Trends in Biochemical Sciences, 32, 95–100. 10.1016/j.tibs.2006.12.002 17174552

[acel13615-bib-0032] Ozcelik, S. , Fraser, G. , Castets, P. , Schaeffer, V. , Skachokova, Z. , Breu, K. , Clavaguera, F. , Sinnreich, M. , Kappos, L. , Goedert, M. , Tolnay, M. , & Winkler, D. T. (2013). Rapamycin attenuates the progression of tau pathology in P301S tau transgenic mice. PLoS One, 8, e62459. 10.1371/journal.pone.0062459 23667480 PMC3646815

[acel13615-bib-0033] Patterson, K. R. , Remmers, C. , Fu, Y. , Brooker, S. , Kanaan, N. M. , Vana, L. , Ward, S. , Reyes, J. F. , Philibert, K. , Glucksman, M. J. , & Binder, L. I. (2011). Characterization of prefibrillar Tau oligomers in vitro and in Alzheimer disease. Journal of Biological Chemistry, 286, 23063–23076. 10.1074/jbc.M111.237974 21550980 PMC3123074

[acel13615-bib-0034] Rodriguez, A. , Durán, A. , Selloum, M. , Champy, M. F. , Diez‐Guerra, F. J. , Flores, J. M. , Serrano, M. , Auwerx, J. , Diaz‐Meco, M. T. , & Moscat, J. (2006). Mature‐onset obesity and insulin resistance in mice deficient in the signaling adapter p62. Cell Metabolism, 3, 211–222. 10.1016/j.cmet.2006.01.011 16517408

[acel13615-bib-0035] Sahara, N. , DeTure, M. , Ren, Y. , Ebrahim, A. S. , Kang, D. , Knight, J. , Volbracht, C. , Pedersen, J. T. , Dickson, D. W. , Yen, S.‐H. , & Lewis, J. (2013). Characteristics of TBS‐extractable hyperphosphorylated tau species: Aggregation intermediates in rTg4510 mouse brain. Journal of Alzheimer's Disease, 33, 249–263. 10.3233/JAD-2012-121093 PMC3514650 22941973

[acel13615-bib-0036] Santacruz, K. , Lewis, J. , Spires, T. , Paulson, J. , Kotilinek, L. , Ingelsson, M. , Guimaraes, A. , DeTure, M. , Ramsden, M. , McGowan, E. , Forster, C. , Yue, M. , Orne, J. , Janus, C. , Mariash, A. , Kuskowski, M. , Hyman, B. , Hutton, M. , & Ashe, K. H. (2005). Tau suppression in a neurodegenerative mouse model improves memory function. Science, 309, 476–481. 10.1126/science.1113694 16020737 PMC1574647

[acel13615-bib-0037] Schaeffer, V. , Lavenir, I. , Ozcelik, S. , Tolnay, M. , Winkler, D. T. , & Goedert, M. (2012). Stimulation of autophagy reduces neurodegeneration in a mouse model of human tauopathy. Brain, 135, 2169–2177. 10.1093/brain/aws143 22689910 PMC3381726

[acel13615-bib-0038] Scott, I. S. , & Lowe, J. S. (2007). The ubiquitin‐binding protein p62 identifies argyrophilic grain pathology with greater sensitivity than conventional silver stains. Acta Neuropathologica, 113, 417–420. 10.1007/s00401-006-0165-6 17146637

[acel13615-bib-0039] Shi, H.‐R. , Zhu, L.‐Q. , Wang, S.‐H. , Liu, X.‐A. , Tian, Q. , Zhang, Q. , Wang, Q. , & Wang, J.‐Z. (2008). 17beta‐estradiol attenuates glycogen synthase kinase‐3beta activation and tau hyperphosphorylation in Akt‐independent manner. Journal of Neural Transmission (Vienna), 115, 879–888. 10.1007/s00702-008-0021-z 18217188

[acel13615-bib-0040] Shin, J. (1998). P62 and the sequestosome, a novel mechanism for protein metabolism. Archives of Pharmacal Research, 21, 629–633. 10.1007/BF02976748 9868528

[acel13615-bib-0041] Tanemura, K. , Murayama, M. , Akagi, T. , Hashikawa, T. , Tominaga, T. , Ichikawa, M. , Yamaguchi, H. , & Takashima, A. (2002). Neurodegeneration with tau accumulation in a transgenic mouse expressing V337M human tau. Journal of Neuroscience, 22, 133–141. 10.1523/JNEUROSCI.22-01-00133.2002 11756496 PMC6757582

[acel13615-bib-0042] Ward, S. M. , Himmelstein, D. S. , Ren, Y. , Fu, Y. , Yu, X.‐W. , Roberts, K. , Binder, L. I. , & Sahara, N. (2014). TOC1: A valuable tool in assessing disease progression in the rTg4510 mouse model of tauopathy. Neurobiology of Diseases, 67, 37–48. 10.1016/j.nbd.2014.03.002 PMC4055868 24631720

[acel13615-bib-0043] Xu, Y. , Zhang, S. , & Zheng, H. (2019). The cargo receptor SQSTM1 ameliorates neurofibrillary tangle pathology and spreading through selective targeting of pathological MAPT (microtubule associated protein tau). Autophagy, 15, 583–598. 10.1080/15548627.2018.1532258 30290707 PMC6526869

[acel13615-bib-0044] Yoshiyama, Y. , Higuchi, M. , Zhang, B. , Huang, S.‐M. , Iwata, N. , Saido, T. C. , Maeda, J. , Suhara, T. , Trojanowski, J. Q. , & Lee, V. M. (2007). Synapse loss and microglial activation precede tangles in a P301S tauopathy mouse model. Neuron, 53, 337–351. 10.1016/j.neuron.2007.01.010 17270732

[acel13615-bib-0045] Zaffagnini, G. , Savova, A. , Danieli, A. , Romanov, J. , Tremel, S. , Ebner, M. , Peterbauer, T. , Sztacho, M. , Trapannone, R. , Tarafder, A. K. , Sachse, C. , & Martens, S. (2018). p62 filaments capture and present ubiquitinated cargos for autophagy. EMBO Journal, 37, e98308. 10.15252/embj.201798308 29343546 PMC5830917

[acel13615-bib-0046] Zhang, M. R. , Kumata, K. , Maeda, J. , Yanamoto, K. , Hatori, A. , Okada, M. , Higuchi, M. , Obayashi, S. , Suhara, T. , & Suzuki, K. (2007). 11C‐AC‐5216: A novel PET ligand for peripheral benzodiazepine receptors in the primate brain. Journal of Nuclear Medicine, 48, 1853–1861. 10.2967/jnumed.107.043505 17978354

