# Supplementary material for: Central role for p62/SQSTM1 in the elimination of toxic tau species in a mouse model of tauopathy
Source: Aging Cell. 2022 Jun 5;21(7):e13615. doi: 10.1111/acel.13615 (PMC9282839; doi:10.1111/acel.13615)
Supplement: Supplementary file 1 — Supplementary Material [file ACEL-21-e13615-s001.pdf]

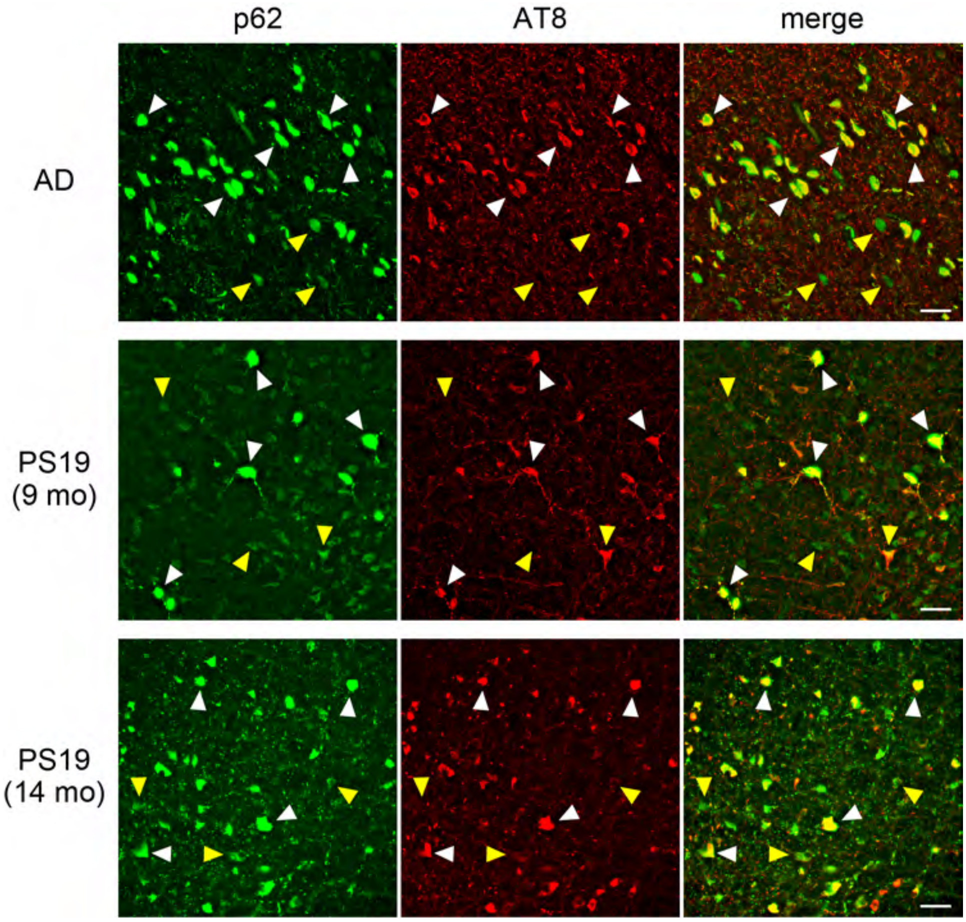

Supplemental figure 1. Double-labeling of phosphorylated tau and p62 in the brain sections derived from tauopathy patients and PS19 mice

Brain sections of hippocampus derived from AD patients and brainstems of 9- and 14-month-old PS19 mice were immunolabeled with antibodies against p62 (green) and phosphorylated tau (AT8, red). White arrowheads represent merging of p62 and phosphorylated tau immunoreactivity. Yellow arrowheads represent the punctate immunoreactivity of p62 in neuronal somas without accumulation of phosphorylated tau in the hippocampus of AD patients and the brainstems of PS19 mice. Scale bars, 50 μm.

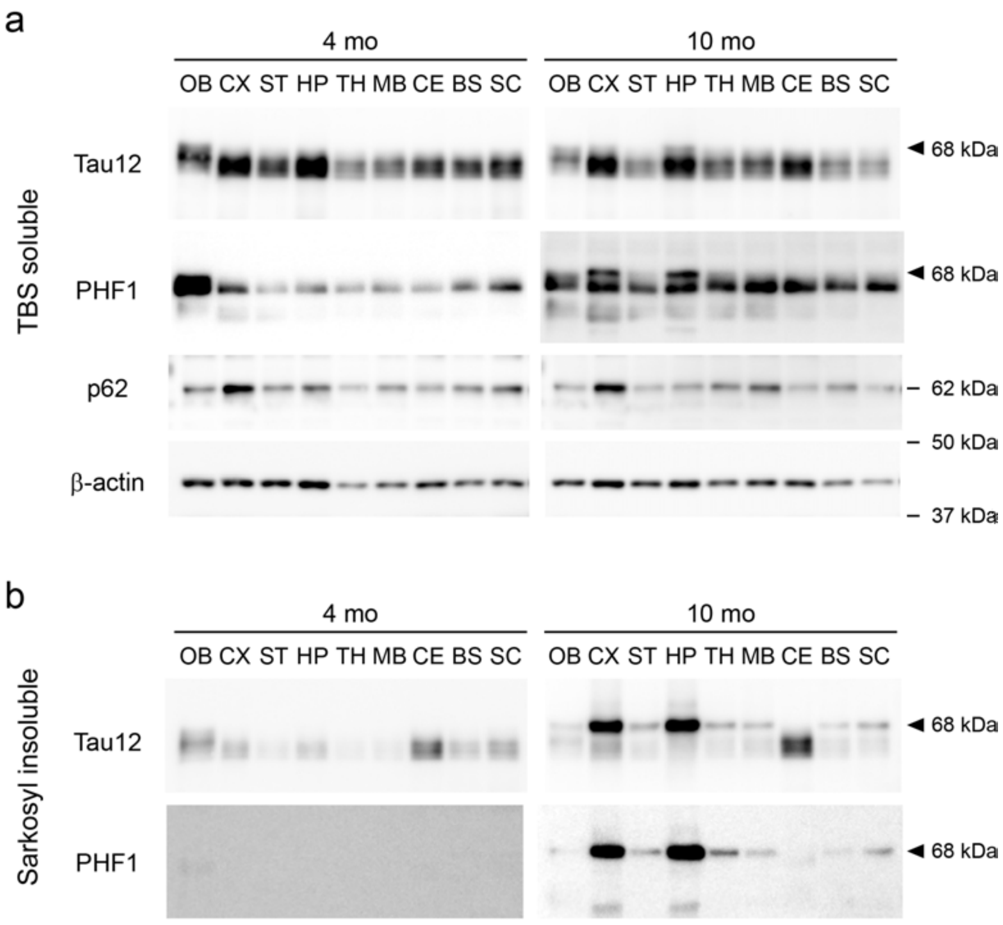

Supplemental figure 2. Brain regional distributions of human P301S tau and p62 in PS19 mice

After dissecting mouse brains, brain regions were separated into olfactory bulb (OB), cerebral cortex (CX), striatum (ST), hippocampus (HP), thalamus (TH), midbrain (MB), cerebellum (CE), brainstem (BS), and spinal cord (SC). TBS soluble and sarkosyl-insoluble fractions were collected from each brain region. (**a**) TBS soluble fractions from 4-month-old and 10-month-old PS19 mice were examined for anti-tau (Tau12 and PHF1), anti-p62, and anti-β-actin antibodies. (**b**) Sarkosyl-insoluble fractions from 4-month-old and 10-month-old PS19 mice were examined for anti-tau (Tau12 and PHF1) antibodies. Arrowheads indicate 68 kDa bands of hyperphosphorylated tau.

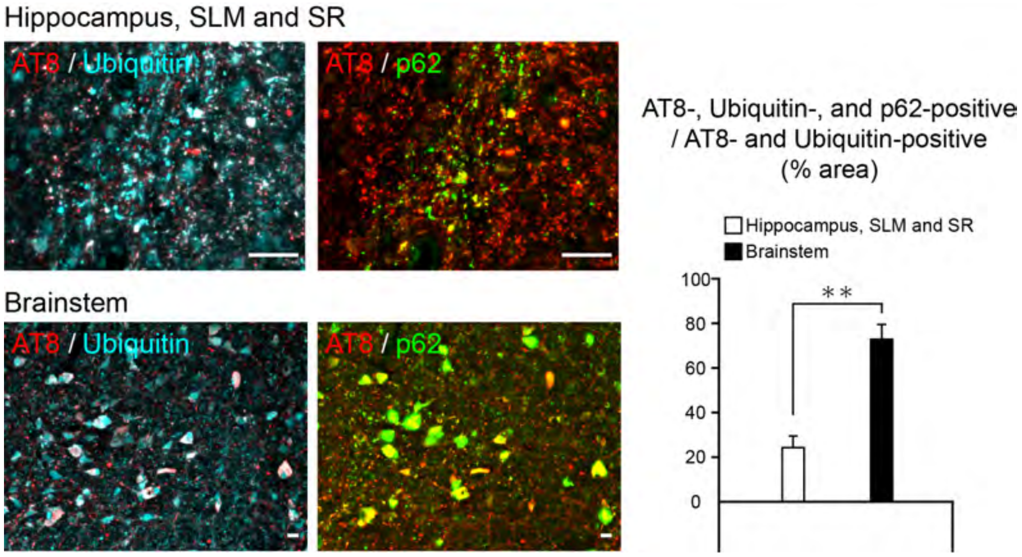

Supplemental figure 3. Triple-labeling of phosphorylated tau, ubiquitin, and p62 in the brain section derived from PS19 mice

(**a**) Stratum lacunosum-moleculare (SLM) and stratum radiatum (SR) of hippocampus and brainstem from 14-month-old PS19 mice were triple-immunolabeled with AT8 (red), anti-ubiquitin (blue), and anti-p62 (green) antibodies. Positive areas of fluorescence staining were quantified (n=3, respectively). Scale bars, 20 μm. Data are presented as mean ± SD. Group comparisons were performed by Welch's t-test (*P<0.05, **P<0.005).

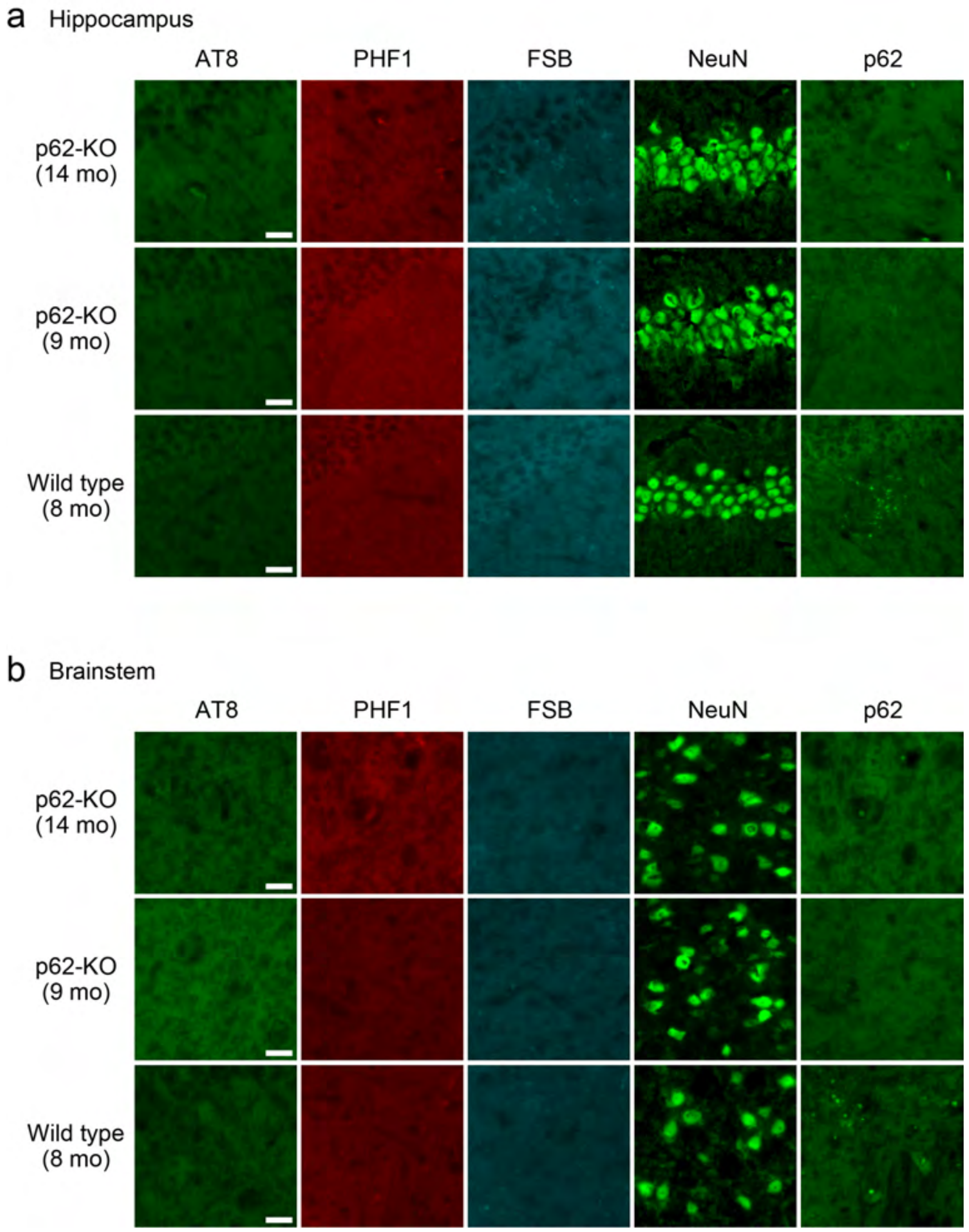

Supplemental figure 4. Histological observation of pathological tau, NeuN and p62 in mouse hippocampus and brainstem

(**a**, **b**) Hippocampal (**a**) and brainstem (**b**) sections from p62-KO (14- and 9-month-old (mo)) and wild type (8-month-old) mice were immunolabeled with AT8 (green), PHF1 (red), anti-NeuN (green), and anti-p62 (green) antibodies and stained with FSB fluorescence dye (blue). Scale bars, 20 μm.

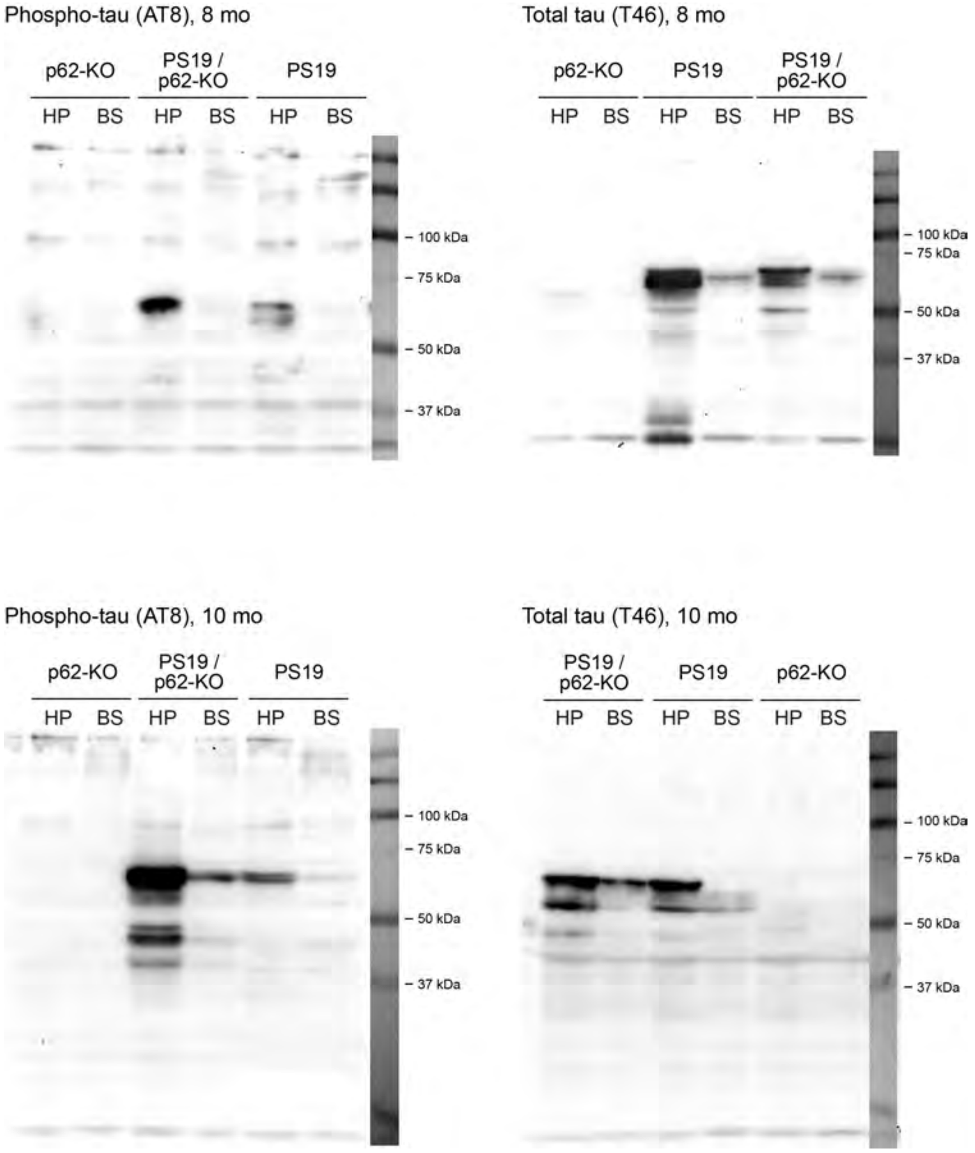

Supplemental figure 5. Accumulation levels of phosphorylated tau in the hippocampus and brainstem of p62-KO, PS19 and PS19/p62-KO mice

Total extracts from the hippocampus (HP) and brainstem (BS) of 8- and 10-month-old (mo) p62-KO, PS19 and PS19/p62-KO mice were separated by SDS-PAGE and blotted with AT8 and T46 antibodies.

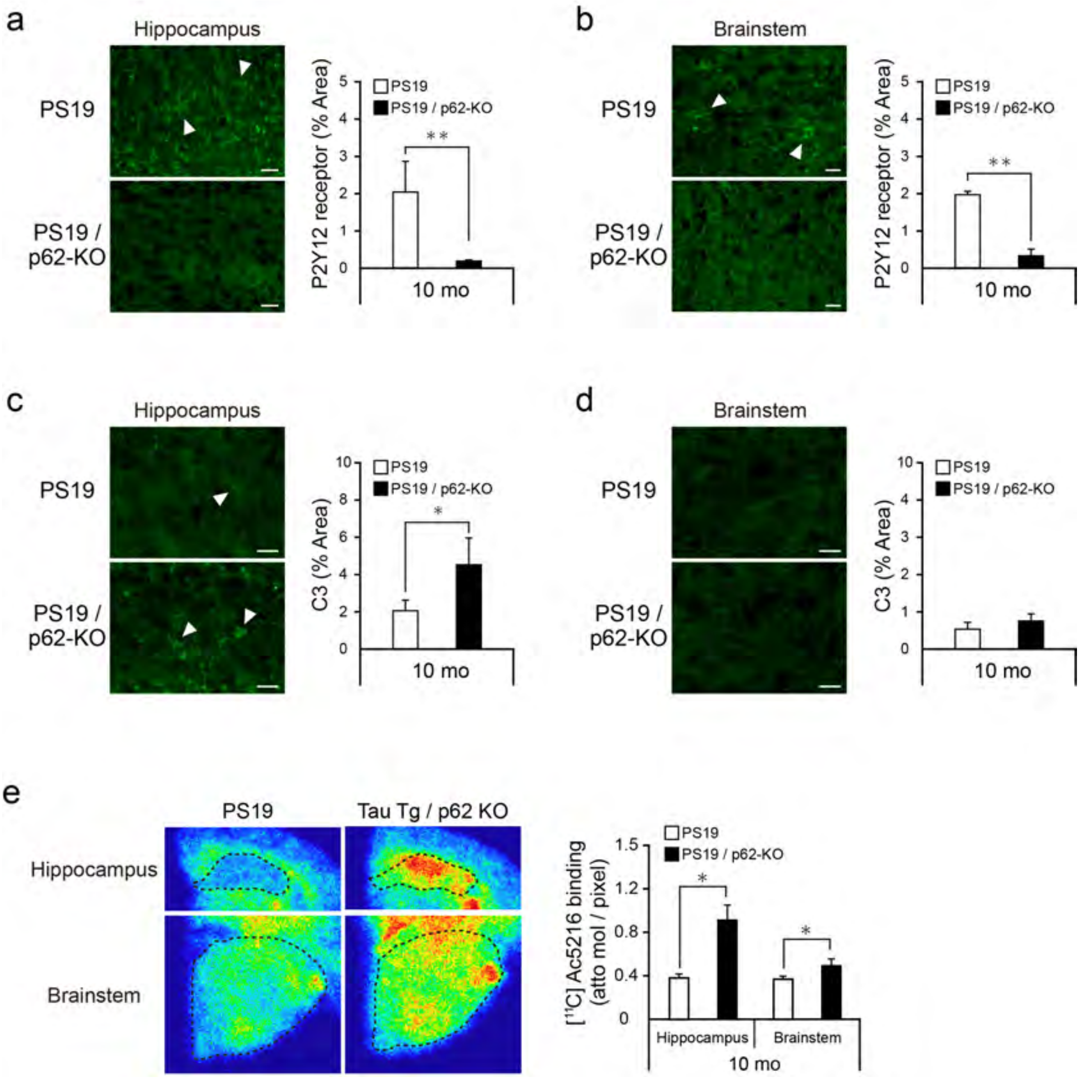

Supplemental figure 6. Enhancement of neuroinflammation in PS19 mice by genetic inactivation of p62

(**a**) The hippocampi from 10-month-old PS19 and PS19/p62-KO mice were immunostained with anti-P2Y12 receptor antibody. Arrowheads show P2Y12 receptor-positive signal. Positive areas of fluorescence staining were quantified (PS19: *n*=3, PS19/p62-KO: *n*=5). (**b**) The brainstems from 10-month-old PS19 and PS19/p62-KO mice were immunostained with anti-P2Y12 receptor antibody. Arrowheads show P2Y12 receptor-positive signal. Positive areas of fluorescence staining were quantified (*n*=5, respectively). (**c**) The hippocampi from 10-month-old PS19 and PS19/p62-KO mice were immunostained with anti-C3 antibody. Arrowheads show C3-positive signal. Positive areas of fluorescence staining were quantified (PS19: *n*=3, PS19/p62-KO:

n=5). (**d**) The brainstems from 10-month-old PS19 and PS19/p62-KO mice were immunostained with anti-C3 antibody. Positive areas of fluorescence staining were quantified (*n*=5, respectively). (**e**) Glial response was quantified by autoradiography for TSPO with [^{11}C]Ac5216 in the hippocampus and brainstem of 10-month-old PS19 and PS19/p62-KO mice (n=5, respectively). Signal intensities of autoradiography were determined using Multi Gauge. In the hippocampus and brainstem, TSPO was markedly upregulated in PS19/p62-KO mice compared with PS19 mice. Scale bars, 20 μm. Data are presented as mean ± SD. Group comparisons were performed by Welch's *t*-test (*P<0.05, **P<0.005).

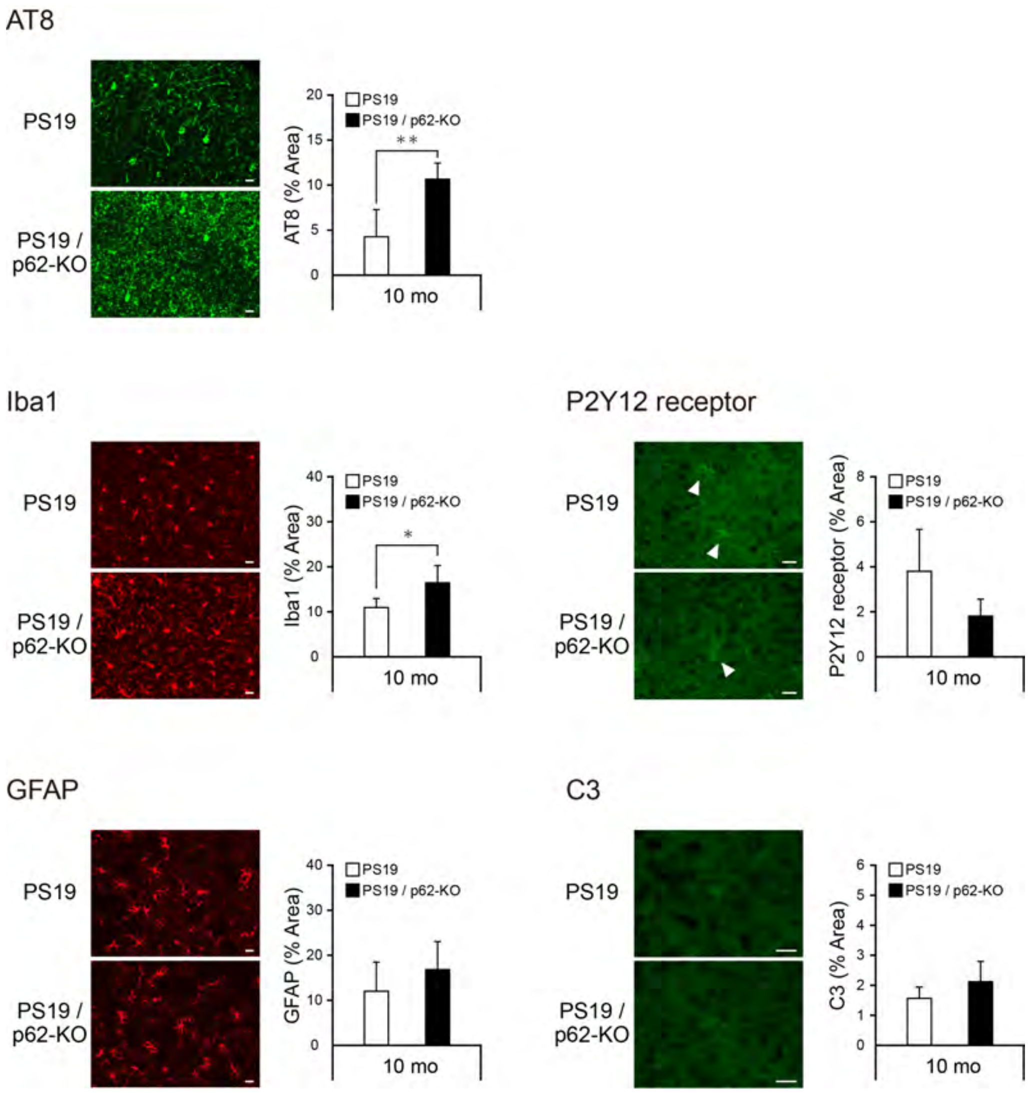

Supplemental figure 7. Enhancement of tau pathologies in the cortex of PS19 mice by genetic inactivation of p62

The cortices from 10-month-old PS19 and PS19/p62-KO mice were immunostained with AT8, Iba1, P2Y12 receptor, GFAP, and C3 antibody. Positive areas of fluorescence staining were quantified (PS19: n=4, PS19/p62-KO: n=5). Scale bars, 20 µm. Data are presented as mean ± SD. Group comparisons were performed by Welch's t-test (*P<0.05, **P<0.005).

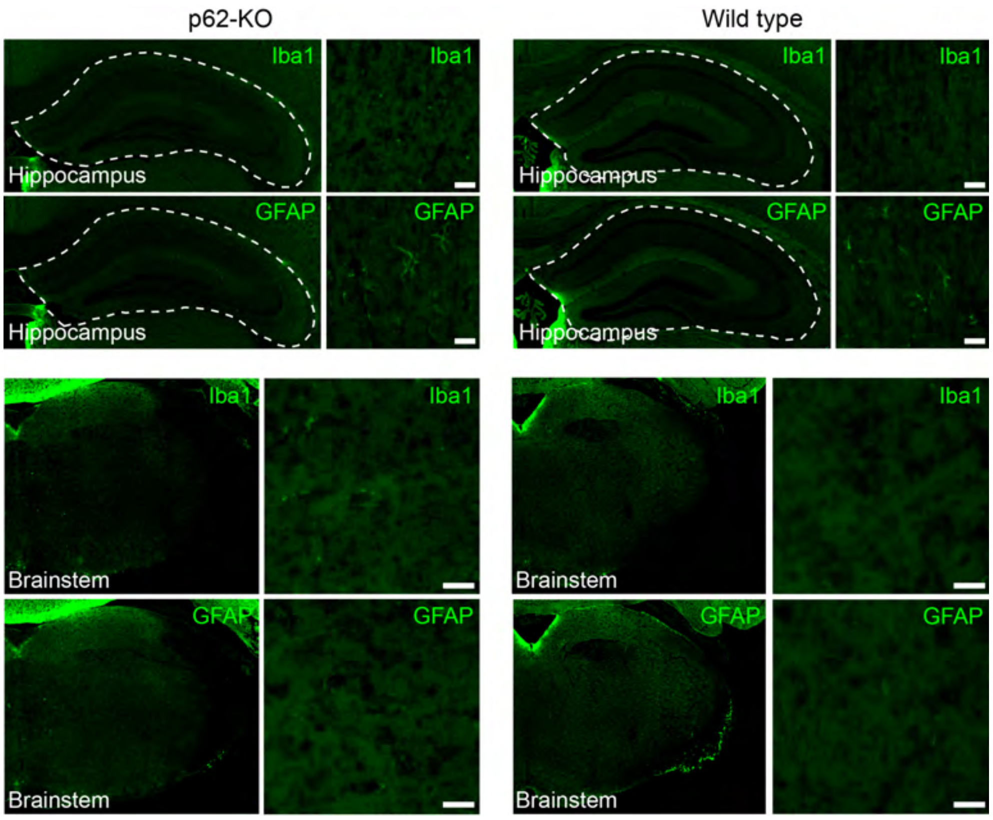

Supplemental figure 8. Immunofluorescence staining of microglia and astrocytes in p62-KO mouse brains

Brain sections of p62-KO (left) and wild type (right) mice at 9 months of age were immunostained with glial markers. Upper panels show hippocampal region labeled with anti-Iba1 antibody or anti-GFAP antibody. Lower panels show brainstem region labeled with anti-Iba1 antibody or anti-GFAP antibody. High- and low-magnification views are side-by-side. Scale bars in high-magnification views are 20 μm.

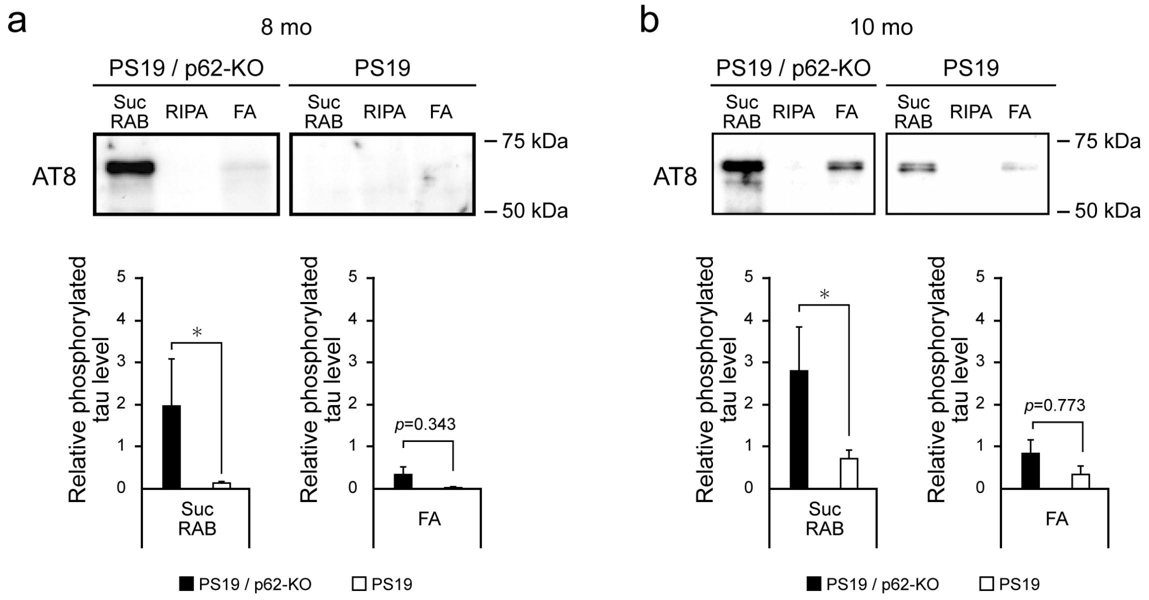

Supplemental figure 9. Biochemical properties of tau proteins extracted from PS19 and PS19/p62-KO mouse hippocampi

(**a**) Representative western blot images of fractionated samples from mouse hippocampus. Tissue homogenates from 8-month-old (mo) PS19/p62-KO and PS19 mouse hippocampi were separated into 1M sucrose-RAB, RIPA and FA fractions. Samples were run on SDS-PAGE, and immunoblotted with AT8 antibody. Immunoblot signals were quantified (n=4, respectively). *P<0.05 by Mann-Whitney U test. (**b**) Representative western blot images of fractionated samples from mouse hippocampus. Tissue homogenates from 10-month-old (mo) PS19/p62-KO and PS19 mouse hippocampi were separated into 1M sucrose-RAB, RIPA and FA fractions. Samples were run on SDS-PAGE, and immunoblotted with AT8 antibody. Immunoblot signals were quantified (n=4, respectively). *P<0.05 by Mann-Whitney U test. Data are presented as mean ± SEM.

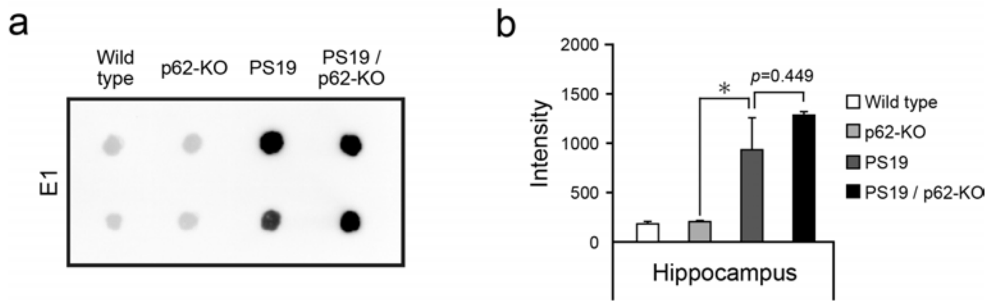

Supplemental figure 10. Dot blot analysis of tau protein in 1M sucrose-RAB fractions from mouse hippocampus

(**a**) Representative dot blot image of hippocampal extracts labeled by E1 antibody. Equal volumes of 1M sucrose-RAB fractions recovered from the hippocampi of 10-month-old wild type, p62-KO, PS19, and PS19/p62-KO mice were spotted on nitrocellulose membranes and incubated in E1 antibody. Primary antibodies were detected by HRP-conjugated anti-rabbit IgG antibody and enhanced chemiluminescence method. (**b**) Immunoblot signals were quantified (*n*=3, respectively). Group comparisons were performed by one-way ANOVA followed by Tukey's HSD test. Data are presented as mean ± SEM.

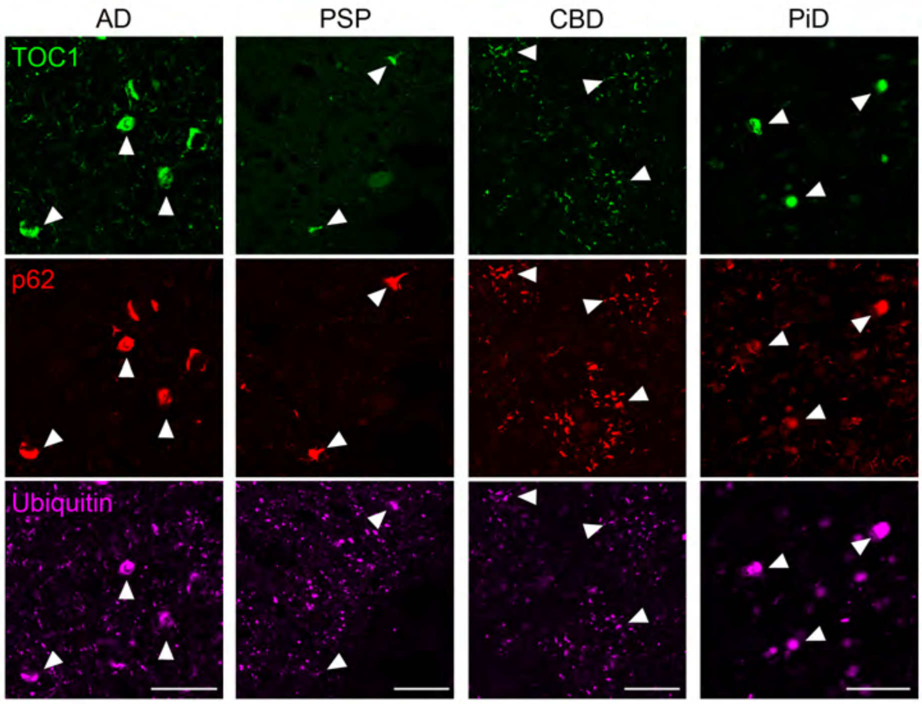

Supplemental figure 11. Accumulation of oligomeric tau species in human tauopathies

The hippocampus from AD, striatum from PSP, frontal cortex from CBD and PiD patients were triple-immunolabeled with TOC1 (green), anti-ubiquitin (blue), and anti-p62 (red) antibodies. Arrowheads indicate triple-labeling with TOC1, ubiquitin, and p62 antibodies. Scale bars, 50 μm.

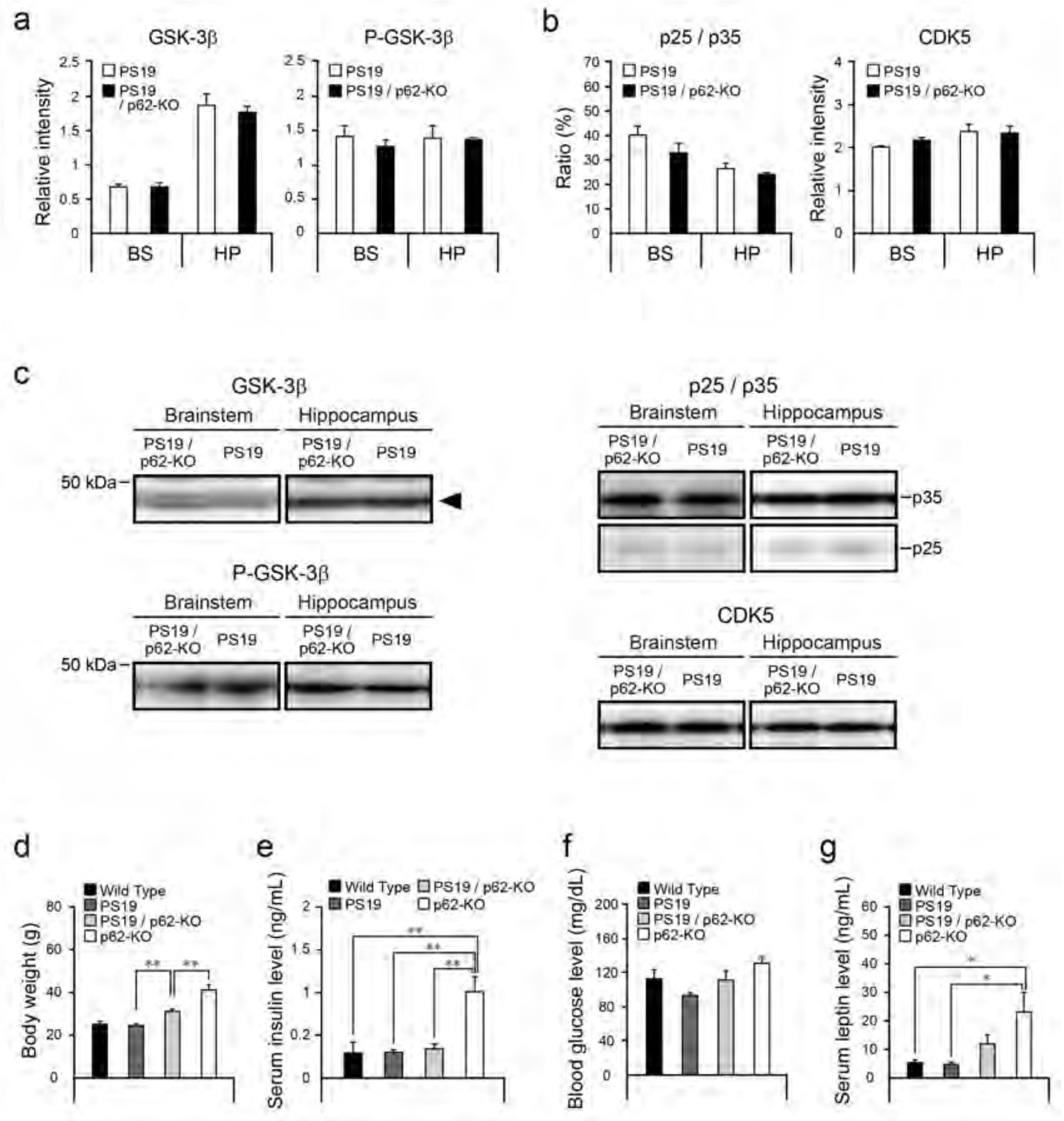

Supplemental figure 12. Tau kinase activities and circulating levels of typical obesity-related factors

(**a**, **b**) Total extracts from the brainstems and hippocampi of 8-month-old PS19 and PS19/p62-KO mice were run on SDS-PAGE and immunoblotted with anti-GSK-3β (**a**, left), anti-phosphorylated GSK-3β (**a**, right), anti-p25/p35 (**b**, left), or anti-CDK5 (**b**, right) antibodies. Immunoblot signals were quantified (*n*=4, respectively). Group comparisons were performed by Welch's *t*-test at each region. (**c**) Representative western blot images of GSK-3β, phosphorylated GSK-3β, p25/p35, and CDK5. Primary antibodies were detected by HRP-conjugated anti-IgG antibodies and enhanced

chemiluminescence method. (**d**) Body weights were measured in wild type ($n=10$), PS19 ($n=10$), PS19/p62-KO ($n=10$), and p62-KO ($n=8$) mice at 8 months of age (group $F(3, 34)=35.906$, $p<0.001$, PS19/p62-KO vs p62-KO $p<0.001$). *$P<0.05$ by one-way ANOVA followed by Tukey's HSD test. (**e**) Levels of insulin in serum from 8-month-old wild type, PS19, PS19/p62-KO, and p62-KO mice were measured by ELISA ($n=5$, respectively; group $F(3, 14)=9.326$, $p=0.0012$, PS19/p62-KO vs PS19 $p=0.7349$). **$P<0.005$ by one-way ANOVA followed by Tukey's HSD test. (**f**) Blood glucose levels were measured in 8-month-old wild type, PS19, PS19/p62-KO, and p62-KO ($n=5$, respectively; group $F(3, 16)=3.065$, $p=0.058$, PS19/p62-KO vs PS19 $p=0.515$). Group comparisons were performed by one-way ANOVA. (**g**) Levels of leptin in serum from 8-month-old wild type, PS19, PS19/p62-KO, and p62-KO mice were measured by ELISA ($n=5$, respectively; group $F(3, 16)=5.519$, $p=0.009$, PS19/p62-KO vs PS19 $p=0.514$, PS19/p62-KO vs wild type $p=0.584$, p62-KO vs PS19 $p=0.0121$, p62-KO vs wild type $p=0.0153$). *$P<0.05$ by one-way ANOVA followed by Tukey's HSD test. Data are presented as mean ± SEM.

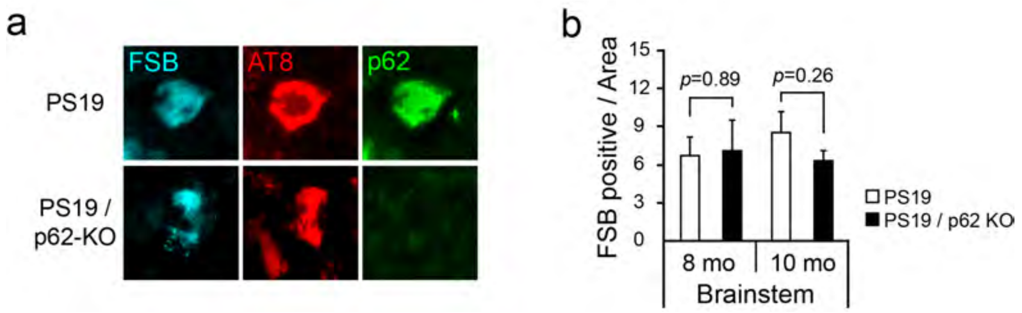

Supplemental figure 13. Quantitative analysis of mature tau inclusions in PS19 and PS19/p62-KO mouse brainstem

(a) Representative triple-staining images of intraneuronal tau inclusions. The brainstems from 8-month-old PS19 (upper panels) and PS19/p62-KO (lower panels) mice were immunostained with AT8 and anti-p62 antibodies, and fluorescence stained with FSB. (b) FSB-positive cell numbers in brainstems of 8- and 10-month-old (mo) PS19 and PS19/p62-KO mice (n=5, respectively) were quantified. Group comparisons were performed by Welch's *t*-test at each age.

Supplementary table. Antemortem clinical diagnosis and neuropathologic description of postmortem human brain tissues

Case	Pathologic diagnosis	Clinical diagnosis	Age	Sex	Braak stage	CERAD plaque score
AD	AD	AD probable	89	Female	V/VI	C
PSP	PSP	PSP	67	Male	V/VI	0
CBD	CBD	FTLD-NOS	59	Female	0	0
PiD	PiD	FTLD-bvFTD	71	Male	0	0

AD, Alzheimer's disease; PSP, progressive supranuclear palsy; CBD, corticobasal degeneration; FTLD-NOS, frontotemporal lobar degeneration not otherwise specified; PiD, Pick's disease; FTLD-bvFTD, frontotemporal lobar degeneration behavioral variant frontotemporal dementia
